# The evolutionary dynamics of variant antigen genes in *Babesia* reveal a history of genomic innovation underlying host–parasite interaction

**DOI:** 10.1093/nar/gku322

**Published:** 2014-05-05

**Authors:** Andrew P. Jackson, Thomas D. Otto, Alistair Darby, Abhinay Ramaprasad, Dong Xia, Ignacio Eduardo Echaide, Marisa Farber, Sunayna Gahlot, John Gamble, Dinesh Gupta, Yask Gupta, Louise Jackson, Laurence Malandrin, Tareq B. Malas, Ehab Moussa, Mridul Nair, Adam J. Reid, Mandy Sanders, Jyotsna Sharma, Alan Tracey, Mike A. Quail, William Weir, Jonathan M. Wastling, Neil Hall, Peter Willadsen, Klaus Lingelbach, Brian Shiels, Andy Tait, Matt Berriman, David R. Allred, Arnab Pain

**Affiliations:** 1 Department of Infection Biology, Institute of Infection and Global Health, University of Liverpool, Liverpool Science Park Ic2, 146 Brownlow Hill, Liverpool L3 5RF, UK; 2 Pathogen Genomics Group, Wellcome Trust Sanger Institute, Wellcome Trust Genome Campus, Hinxton, Cambridge CB10 1SA, UK; 3 Department of Functional and Comparative Genomics, Institute of Integrative Biology, University of Liverpool, Crown Street, Liverpool L69 7ZB, UK; 4 Computational Bioscience Research Center, King Abdullah University of Science and Technology, Thuwal 23955-6900, Saudi Arabia; 5 Laboratorio de Inmunología y , EEA-INTA CC 22, 2300 Rafaela Santa Fe, Argentina; 6 Centro Nacional de Investigaciones Agropecuarias, Instituto de Biotecnología INTA, Buenos Aires, Argentina; 7 Bioinformatics Laboratory, Structural and Computational Biology Group, International Centre for Genetic Engineering and Biotechnology, Aruna Asaf Ali Marg, New Delhi 110 067, India; 8 Department of Agriculture, Fisheries and Forestry, Biosecurity Sciences Laboratory, 39 Kessels Road, Coopers Plains, Queensland 4108, Australia; 9 UMR1300 INRA/Oniris Biology, Epidemiology and Risk Analysis in Animal Health, BP 40706, F-44307 Nantes, France; 10 FG Parasitologie, Philipps Universität Marburg, Karl von Frisch Strasse 8, 35043 Marburg, Germany; 11 Institute of Biodiversity, Animal Health and Comparative Medicine, University of Glasgow, 464 Bearsden Road, Glasgow G61 1QH, UK; 12 Department of Infectious Diseases and Pathology, and Genetics Institute, University of Florida, PO Box 110880, 2015 SW 16th Avenue, Gainesville FL 33611-0880, USA

## Abstract

*Babesia* spp. are tick-borne, intraerythrocytic hemoparasites that use antigenic variation to resist host immunity, through sequential modification of the parasite-derived variant erythrocyte surface antigen (VESA) expressed on the infected red blood cell surface. We identified the genomic processes driving antigenic diversity in genes encoding VESA (*ves1*) through comparative analysis within and between three *Babesia* species, (*B. bigemina*, *B. divergens* and *B. bovis*). *Ves1* structure diverges rapidly after speciation, notably through the evolution of shortened forms (*ves2*) from 5′ ends of canonical *ves1* genes. Phylogenetic analyses show that *ves1* genes are transposed between loci routinely, whereas *ves2* genes are not. Similarly, analysis of sequence mosaicism shows that recombination drives variation in *ves1* sequences, but less so for *ves2*, indicating the adoption of different mechanisms for variation of the two families. Proteomic analysis of the *B. bigemina* PR isolate shows that two dominant VESA1 proteins are expressed in the population, whereas numerous VESA2 proteins are co-expressed, consistent with differential transcriptional regulation of each family. Hence, VESA2 proteins are abundant and previously unrecognized elements of *Babesia* biology, with evolutionary dynamics consistently different to those of VESA1, suggesting that their functions are distinct.

## INTRODUCTION

Antigenic variation in pathogens is an adaptation to protective host immunity. It describes the serial replacement of variant antigens situated on the pathogen surface during an infection, causing the negation of immune responses mounted to the preceding antigen. Ultimately, the host cannot gain effective immunity and so suffers persistent infection and recurrent disease. Antigenic variation is a tangible expression of the coevolutionary arms race between hosts and pathogens and has evolved on several occasions in diverse lineages (1). This includes the etiological agents of prominent diseases of humans and animals, such as malaria (2), trypanosomiasis (3,4), influenza (5), bacterial meningitis (6) and anaplasmosis (7). Since antigenic variation is fundamental to circumventing the immune response, it plays a central role in pathogenesis and virulence and is one of the main obstacles to developing effective vaccines (8–10).

This study investigates antigenic variation in *Babesia* spp., apicomplexan hemoparasites that cause a tick-borne disease in animals and occasionally in people (11). Babesiosis has a global distribution, and while found predominantly in tropical and sub-tropical regions, it frequently reaches far into temperate regions (12). Bovine babesiosis has a significant negative impact on livestock productivity in both developed and developing countries. While most infections are asymptomatic, mild infections can cause fever and diarrhoea while severe infections, of the type caused by *Babesia bovis* in cattle, lead to acute haemolytic anaemia and may trigger a cerebral disease that is likely inflammatory in origin and almost invariably terminal. Here we examine two additional species that cause babesiosis in livestock, *Babesia bigemina* (13) and *Babesia divergens* (14). These three species are members of the *Babesia sensu stricto* clade and, of the three, *B. bigemina* and *B. divergens* are most closely related (15). Babesiosis in humans is uncommon; however, *B. divergens* infections are increasingly reported in immuno-compromized or splenectomized patients, often causing death (16). *Babesia microti* is another human parasite but will not be considered here since, despite its generic name and the presence of three *ves*-like genes in its genome (17), it is relatively distant from *Babesia sensu stricto* in phylogenetic terms and offers limited value as an out-group in this study (15).

*Babesia* spp. infections begin with invasion of host red blood cells and continue through cycles of intra-erythrocytic re-invasion. Secreted parasite proteins are present on the surface of infected red blood cells (IRBC). Variation in these epitopes first indicated that serological responses to *B. bovis* infections are isolate-specific and do not provide lasting immunity (18). Antigenic variation was directly inferred from successive waves of gain and loss of immuno-reactivity to the major expressed IRBC surface antigen, and changes in antigen size, over the course of a clonal infection (19). The proteins responsible for these responses in *B. bovis* are the heterodimeric variant erythrocyte surface antigen (VESA), encoded by the multi-copy *ves1α* and *ves1β* gene families in *B. bovis* (20,21). *ves1* genes are expressed from specific sites known as loci of active transcription (LAT) that typically contain a *ves1α* gene and a *ves1β* gene in divergent orientation (22). *ves1* gene expression is regulated by bidirectional promoters within the LAT intergenic region (23) and involves localized chromatin remodeling (24). This precise regulatory mechanism is thought to ensure monoallelic expression, in which only the ‘active’ *ves1* gene within the LAT is expressed while all other loci are silenced; and recent analysis of *ves1* complementary deoxyribonucleic acid (cDNA) in *B. bovis* C9.1 supported this view (25).

The *ves1* gene repertoire of *B. bovis* has been established from its complete genome sequence (26). This family contains 72 *ves1α*, 43 *ves1β* and four atypical genes that do not conform to either structure; 66 *ves1* (55%) are arranged in divergent orientation, i.e. potential LATs. These loci are distributed throughout the chromosomes, and overall, 75% of *ves1* genes are found in the interstitial chromosome regions, rather than showing any positional bias towards sub-telomeres. LATs also tend to contain a third locus, the small open reading frame (*smORF*) genes (26), whose function is unknown. *Ves1α* and *ves1β* genes have different gene architectures; canonical *ves1α* genes consist of three exons and two short introns; *ves1β* genes vary widely in intron number and length, with up to 11 introns (20,22,26). Due to the relative simplicity of the *ves1α* gene structure and lack of structural variation between paralogs relative to *ves1β*, it was suggested that *ves1α* may have originated through the retrotranscription of an incompletely spliced *ves1β* gene transcript (22).

The ability of pathogens to employ antigenic variation in the long-term depends on the generation of novel variant antigens through evolutionary change. Genomic research in two models of antigenic variation, *var* genes in the malaria parasite *Plasmodium falciparum* (27–29) and *vsg* genes in the African trypanosome *Trypanosoma brucei* (30,31), have begun to explain how variant antigens change over evolutionary time, and how their dynamics differ from ‘ordinary’ genes (32). By taking a similar approach, we can examine how antigenic diversity is generated in *Babesia*. The evolution of variant antigen genes is rapid; gene duplication is more frequent and sequence divergence faster relative to the genomic background (27,33). Rapid change is due in part to frequent gene conversion, which is a strategy independently evolved by many pathogens to promote antigenic diversity (34). Phylogenetic comparisons of both *var* and *vsg* show that rapid change results in species-specific gene repertoires and a lack of orthology [i.e. genes from different species form mutually exclusive clades (31,35)], as well as structural differentiation of the repertoire for life stage or disease stage-specific expression (36,37), and the derivation of novel, invariant genes that appear to acquire new functions (30,31).

Our understanding of *ves1* gene diversity and antigenic variation is largely based on the analysis of *B. bovis*. In this study, our aim is to characterize the variant antigen repertoires in multiple *Babesia* species using comparative genomics and, to identify how variant antigens originated, how repertoires in other *Babesia* spp. vary from the model based on *B. bovis*, and to examine the evolutionary processes that generate antigenic diversity on a population scale. We have sequenced the genomes of several strains of *B. bigemina* and *B. divergens*. Our results show that, while *ves1* genes are widespread, primary structure and genomic repertoire varies substantially, such that features in *B. bovis* like heterodimerism and organization into LATs, may not occur in other species. This study reveals a history of constant innovation in *Babesia* genomes with respect to genes implicated in antigenic variation, and, in so doing, we have discovered that novel *ves*-like gene families, thought to be secreted, have been independently derived from canonical *ves1* genes in different species. The expression profiles and evolutionary dynamics of these novel *ves*-like genes differ from canonical *ves1* genes, suggesting that they are exposed to fundamentally different functional constraints.

## MATERIALS AND METHODS

### Parasite isolation

The *B. bovis* C9.1 clonal line is a derivative of the Mexico isolate. Its derivation from the MO7 clonal line has been described before (19). The *B. bigemina* BOND clone C7-1 was derived from an Australian isolate in 1991 by the Queensland Department of Agriculture, Fisheries and Forestry. It was cloned by limiting dilution in *in vitro* culture (38) and then stored under liquid nitrogen until 2004 when it was removed and inoculated into a splenectomized *Bos taurus* calf. Parasites were first detected five days after inoculation of the calf and 2 l of peripheral blood was collected after a further two days. Parasites were purified from erythrocytes and white blood cells using a saponin lysis method (39) and then DNA was extracted using phenol–chloroform. The *B. bigemina* JG29 and Puerto Rico (PR) isolates were kind gifts from T.F. McElwain and were maintained in *in vitro* culture for isolation of nucleic acids and proteins. The *B. bigemina* BbiS3P strain was isolated from an 18 months old Braford heifer, chronically infected, in the Salta province, Argentina, in March 2005. The isolation was achieved *in vitro*, by cultivation of heifer's erythrocytes previously washed with buffer VYM. The *B. divergens* 1802A strain was isolated in May 1988 from a cow in the Le Cher department of France with acute babesiosis. The *B. divergens* strain Rouen1987 (40) was a kind gift of H. Vial, Montpellier.

### Cell culture

The *B. bovis* C9.1 clonal line, and *B. bigemina* JG29 and PR isolate parasites were cultivated *in vitro* under microaerophilous stationary phase conditions as described (41), with slight modifications (42). Closed herd bovine blood donors maintained at the University of Florida were used in all work with these strains. All animal procedures were approved by the University of Florida Institutional Animal Care and Use Committee. The *B. bigemina* BbiS3P strain was also cultivated *in vitro* and stored frozen in liquid nitrogen. A culture expansion to achieve ≥50% of parasitized erythrocytes was accomplished to purify merozoites for gDNA extraction. The *B. divergens* 1802A strain was obtained by infecting a single gerbil with the cow blood. *In vitro* culture was initiated from this isolate. After initiation, we cloned the culture by the limiting dilution technique to avoid producing DNA from a mixed parasite population. One clone was selected and expanded *in vitro* immediately. The *B. divergens* Rouen1987 was cultivated in human red blood cells, blood group A+ in 10% human serum of blood group A+. Cultivation was in a 5% O_2_, 5% CO_2_ atmosphere.

### Sample preparation

The *B. bovis* C9.1 and *B. bigemina* JG29 and PR parasites were grown in erythrocytes depleted of contaminating DNA and bovine white blood cells by use of Whatman CF-11 cellulose (43). *B. bigemina* PR-infected erythrocytes were enriched to near homogeneity using Percoll gradients (44). Genomic DNA was isolated as described (45) with spooling at the final step. For protein analysis, cells were washed two times with phosphate-buffered saline, the supernatants were removed and the cells were flash-frozen on dry ice. *B. bigemina* BbiS3P free merozoites were isolated from stroma by using Percoll gradients. Merozoite parasites were pelleted and pellets were lysed at 58°C for 1 h in lysis buffer (0.05 M Tris-HCl pH 8.0, 0.1 M EDTA, 0.1 M NaCl, 2% SDS) with 160 μg of proteinase K (Invitrogen^®^). gDNA was extracted with 1 vol of phenol/chloroform/isoamyl alcohol (Invitrogen^®^), precipitated with ice-cold isopropyl alcohol and washed once with 75% ice-cold ethanol. Pellets were suspended in 50 μl distilled water and were kept at −20°C until use. Genomic DNA was prepared for *B. divergens* 1802A and Rouen1987 from cell lysates of monoclonal cultures using commercial kits (Promega and Qiagen, respectively).

### Genome sequencing and annotation

The *B. bigemina* BOND genome was sequenced using capillary technology to 8X coverage and assembled with Phrap. The genome was manually improved in Gap4. Sequencing errors were corrected using a polymerase chain reaction (PCR) free Illumina library and the iCORN algorithm (46). The *B. divergens* 1802A strain was sequenced using the 454 Titanium platform combining reads from a genomic fragment paired-end library with 3 and 8 kb mate-pair libraries and assembled using Newbler v2.6. Sequencing errors were corrected using additional SOLiD v3 reads. Protein-coding genome annotation for *B. bigemina* BOND and *B. divergens* 1802A were generated by taking a consensus of several *ab initio* gene prediction programs. Annotation from *B. bovis* (26) was used to train Augustus (47), glimmerHMM (48) and SNAP (49). Each of these trained gene finders were then run on the target genomes and this annotation was combined in each case using Jigsaw (50). The annotation was improved manually. Three further *B. bigemina* genomes (PR, BbiS3P and JG29), the *B. bovis* C9.1 genome and the *B. divergens* Rouen1987 genome were sequenced using the Illumina GA platform. The libraries were prepared with the PCR-free protocol and sequenced to 76 read pairs with an insert size of around 350 bp. Illumina reads were assembled with velvet (51). The genomes were improved using the Post Assembly Genome Improvement Toolkit (PAGIT) pipeline (52), applying the contig ordering against the BOND reference using the program ABACUS (53) and the gap-filling step (54). For the *B. bigemina* genome a further gap-filling was performed, using gap-filler (55). Contigs smaller than 1 kb were ignored. Two methods were used to annotate these draft genome sequences and the outcome merged. First, annotation was transferred from the reference genomes (i.e. *B. bovis* T2Bo, *B. divergens* BOND and *B. divergens* 1802A) to additional strains using the Rapid Annotation Transfer Tool (RATT) (56). Genes models with errors were excluded. Second, Augustus (47) was trained with the complete reference gene set to perform *ab initio* annotation.

### Transcriptomic analysis

Strand-specific messenger ribonucleic acid (mRNA) sequencing libraries were prepared from total RNA of *B. bigemina* PR strain (two biological replicates) using TruSeq stranded mRNA Sample Prep Kit LT (Illumina) according to manufacturer's instructions. Briefly, polyA+ mRNA was purified from total RNA using oligo-dT dynabead selection. First strand cDNA was synthesized using randomly primed oligos followed by second strand synthesis where dUTPs were incorporated to achieve strand-specificity. The cDNA was adapter-ligated and the libraries amplified by PCR. Libraries were sequenced in Illumina Miseq with paired-end 150 bp read chemistry. Strand-specific RNA-seq paired-end reads were mapped onto the *B. bigemina* PR genome with TopHat2 (57) with options ‘–library-type = fr-firststranded’ and ‘–no-novel-juncs’. Genes in *B. bigemina* PR orthologous to *B. bigemina* BOND *ves* genes were identified from *B. bigemina* PR gene annotations and their corresponding normalized transcript abundances (FPKM values) were quantified from aligned reads using Cuffdiff2 (58). In case of multiple orthologs in PR for a particular BOND *ves* gene, the orthology with highest FPKM was selected.

### Sample preparation for proteomics

Protein from *B. bigemina* PR cell lysates was dispensed into low protein-binding microcentrifuge tubes (Sarstedt, Leicester, UK) and made up to 160 μl by addition of 25 mM ammonium bicarbonate. The proteins were denatured using 10 μl of 1% (w/v) RapiGest™ (Waters MS Technologies, Manchester, UK) in 25 mm ammonium bicarbonate followed by three cycles of freeze-thaw and two cycles of 10 min sonication in water bath. Sample was then incubated at 80°C for 10 min and reduced with 3 mM dithiothreitol (Sigma-Aldrich, Dorset, UK) at 60°C for 10 min then alkylated with 9 mM iodoacetamide (Sigma-Aldrich, Dorset, UK) at room temperature for 30 min in the dark. Proteomic grade trypsin (Sigma-Aldrich, Dorset, UK) was added at a protein: trypsin ratio of 50:1 and samples incubated at 37°C overnight. In a parallel experiment aiming to maximize protein separation, protein samples were also solubilized and resolved on a NuPAGE^®^ Novex^®^ 4–12% Bis–Tris Gel (Life Technologies Ltd., Paisley, UK) following manufacture's instruction. Four gel slices were excised and each digested with trypsin (Sigma-Aldrich, Dorset, UK).

### Mass spectrometry

Peptide mixtures from both in solution digestion and 1D-sodium dodecyl sulphate-polyacrylamide gel electrophoresis (SDS-PAGE) were analysed by on-line nanoflow liquid chromatography using the nanoACQUITY-nLC system (Waters MS Technologies, Manchester, UK) coupled to an LTQ-Orbitrap Velos (ThermoFisher Scientific, Bremen, Germany) mass spectrometer equipped with the manufacturer's nanospray ion source. The analytical column (nanoACQUITY UPLCTM BEH130 C18 15 cm × 75 μm, 1.7 μm capillary column) was maintained at 35°C and a flow-rate of 300 nl/min. The gradient consisted of 3–40% acetonitrile in 0.1% formic acid for 90 min then a ramp of 40–85% acetonitrile in 0.1% formic acid for 3 min. Full scan MS spectra (*m*/*z* range 300–2000) were acquired by the Orbitrap at a resolution of 30 000. Analysis was performed in data-dependent mode. The top 20 most intense ions from MS1 scan (full MS) were selected for tandem MS by collision induced dissociation and all product spectra were acquired in the LTQ ion trap. Ion trap and Orbitrap maximal injection times were set to 50 and 500 ms, respectively.

### Proteomic analysis

Thermo RAW files were imported into Progenesis LC–MS (version 4.1, Nonlinear Dynamics, UK). Runs were time aligned using default settings and using an auto selected run as reference. Peaks were picked by the software and filtered to include only peaks with a charge state of between +2 and +6. Peptide intensities were normalized against the reference run by Progenesis LC–MS and these intensities are used to highlight differences in protein expression between control and treated samples with supporting statistical analysis (ANOVA and *q*-values) calculated by the Progenesis LC–MS software. Spectral data were transformed to mgf files with Progenesis LC–MS and exported for peptide identification using the Mascot (version 2.3.02, Matrix Science) search engine. Tandem MS data were searched against the predicted protein set of the *B. bigemina* BOND reference genome sequence. Search parameters were as follows: precursor mass tolerance set to 10 ppm and fragment mass tolerance set to 0.8 Da. One missed tryptic cleavage was permitted. Carbamidomethylation (cysteine) was set as a fixed modification and oxidation (methionine) set as a variable modification. Mascot search results were further processed using the machine learning algorithm Percolator. The false discovery rate was <1%. Individual ion scores >13 indicated identity or extensive homology (*P* < 0.05). Results were imported into Progenesis LC–MS as .xml files. At least two unique peptides were required for reporting proteins that were differentially expressed. Results from in solution digestion and gel slices of 1D-SDS PAGE are grouped using ‘Combine analysed fractions’ function in Progenesis LC–MS where statistical analysis are updated.

### Annotation of *ves*-like genes

We searched for *ves*-like and *smORF*-like genes among *B. bigemina* and *B. divergens* translated open reading frames using hidden Markov models (HMM) built using HMMER v3.0 (http://hmmer.janelia.org/) from sequence alignments of *BbovVes1α*, *BbovVes1β* and *BbovSmORF*. Once this had revealed native *ves1* sequences in *B. bigemina* and *B. divergens* (though not *smORF*), these sequences were used in turn to create new hidden Markov models (HMMs) for a second search, which identified *ves2* in each case. Lastly, tBLASTx and BLASTn were applied to the *B. bigemina* and *B. divergens* genome sequences using native *ves1* or *ves2* sequences to identify any unannotated copies. *ves1* structures are highly mutable, and therefore it is inadvisable to simply rely on annotation transfer and sequence homology with known genes in *B. bovis*, which may not adequately capture *ves*-like genes in other genomes. Coding sequences for the *ves*-like genes analysed in this study have been deposited on the Wellcome Trust Sanger Institute FTP site (ftp://ftp.sanger.ac.uk/pub/pathogens/Babesia/).

### Comparative genomics

We used OrthoMCL v2.0.9 (59,60) to examine species-specific genes and gene families with species disparities in copy number. Protein sets from *B. bovis* T2Bo, *B. bigemina* BOND and *B. divergens* 1802A were clustered using OrthoMCL, set to maximize cluster size (i.e. minimized sequence identity requirements for clustering). From these data we calculated the number of genes shared by all three species in single copy (‘conserved’) or as part of gene families with variable copy number (‘semi-conserved’). We also extracted the number of *ves1* homologs and *ves1*-like shortened forms (i.e. *ves2*) in each genome. Finally, we calculated the number of species-specific genes in single or multiple copies. In comparisons of chromosomal rearrangements across species, and in comparisons of conserved *ves1/ves2* loci across strains, we used the Artemis Comparison Tool [ACT (61)] to visualize conservation in gene order.

### Phylogenetic analysis

Translated nucleotide sequences for each sub-family were aligned in ClustalW (62) and then manually edited in BioEdit v7.1.3. (63). A phylogeny of all *ves1*-like genes was estimated by selecting representative samples of BbovVes1α/1β/1γ, *BbigVes1a*/*1b* and *BdivVes1* sequences and creating an 840 character DNA sequence alignment of the conserved C-terminal domain (corresponding to the transmembrane and cytoplasmic domains). The phylogeny was estimated using a GTR+G model in PHYML (64) with 100 non-parametric bootstraps and in MrBayes (65,66) under these settings: Nruns = 4, Ngen = 5 000 000, samplefreq = 500 and default prior distribution. Phylogenies of all sequences were estimated for each sub-family (combining reference and strain sequences) using a GTR+G model in PHYML. Bootstrap proportions were attempted but these were low, as is typical for large alignments of such hypervariable genes. Phylogenies of reference and strain sequences at conserved positions were estimated using the same approach for each species, for use in co-phylogenetic analyses (see below).

### Co-phylogenetic analysis

Phylogenetic reconciliation is a method for resolving topological disparities between two phylogenetic trees that are expected to have the same topology and was initially developed for the comparison of parasite phylogenies with that of their hosts, and of gene family trees with species phylogenies (67,68). Disparities are resolved by positing evolutionary events from a general model of host–parasite or gene family coevolution (i.e. codivergence, duplication, loss and host switching). The statistical significance of topological congruence between two associated trees is typically assessed using permutation tests. Taking each reference genome and one other strain, we identified all *ves*-like loci that were conserved in position and contained a sequence in both genomes. After extracting these sequences, we estimated two phylogenies for each *ves*-like sub-family, one containing ‘reference’ sequences and another ‘strain’ sequences. Since these genes occurred in conserved positions we can assume that they evolved before the separation of reference and strain genomes, and therefore the two phylogenies should look exactly the same in the absence of any transposition between loci after separation (i.e. recombination). The significance of phylogenetic congruence was assessed for each sub-family by permuting the strain tree in Jane 4 (69).

### Recombination analysis

Phylogenetic incompatibility describes the presence of multiple phylogenetic signals within a single sequence alignment and is the historical signature of recombination. The pair-wise homoplasy index (PHI) detects incompatibility between sites and is robust in the presence of rate heterogeneity (70), which might otherwise simulate the effects of recombination. *P* < 0.05 for PHI indicates significant incompatibility between sites within an alignment, consistent with recombination. For each *ves*-like gene family, PHI was calculated for 1000 sequence quartets selected at random from multiple alignments of full-length nucleotide sequences. Sequence triplets were attempted but failed to produce sufficient genetic variation for a viable test in most cases. The proportion of quartet alignments showing significant phylogenetic incompatibility (*P_pi_*) was calculated. A second method for detecting sequence mosaicism was implemented using 3seq (71). 3seq carries out exhaustive comparisons of sequence triplets in a multiple alignment of nucleotide sequences to identify mosaics and returns a *P*-value for each triplet adjusted by a Dunn-Sidak correction for the multiple comparisons made in that run. *P_pi_* was again calculated from the proportion of sequence triplets with *P* < 0.05.

### Data accessibility

Sequence read data have been submitted to the European Nucleotide Archive (http://www.ebi.ac.uk/ena) with the accession numbers ERP000167 and ERP000252. BioProject identifiers (http://www.ncbi.nlm.nih.gov/bioproject/) for the *B. bigemina* BOND and *B. divergens* 1802A reference genomes are PRJEB5046 and PRJNA230984, respectively. Genome sequences for *B. bigemina* strains JG29, BbiS3P and PR, *B. bovis* C9.1 and *B. divergens* Rouen1987 are available from the Wellcome Trust Sanger Institute FTP site (ftp://ftp.sanger.ac.uk/pub/pathogens/Babesia/). Mass spectrometry and proteomic data have been deposited in the ProteomeXchange Consortium (http://proteomecentral.proteomexchange.org) via the PRIDE partner repository (http://www.ebi.ac.uk/pride/) with the dataset identifier PXD000629.

## RESULTS

### Relative structure and content of *Babesia* genome sequences

**Figure 1.**
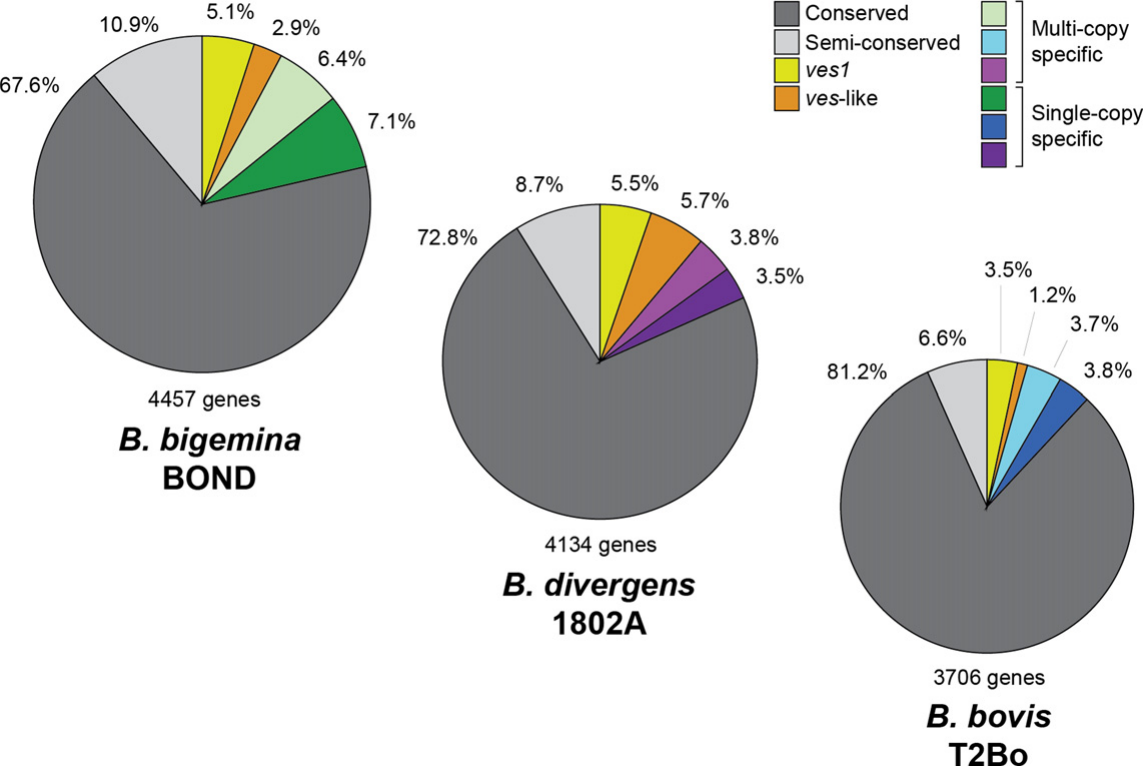
Pie charts showing the classification of predicted coding sequences in three *Babesia* genomes, based on three-way OrthoMCL analysis. Genes with a 1:1:1 distribution are termed ‘conserved’. Genes present in all three species with variable copy number are called ‘semi-conserved’. *ves1* genes in *Babesia bovis* and full-length homologs in other species are represented in yellow. *SmORF* in *B. bovis* and *ves*-like short genes (*ves2*) in other species are represented in orange. The remaining species-specific genes (either single or multi-copy) are represented by green, blue and purple for *Babesia bigemina*, *B. bovis* and *Babesia divergens* respectively.

For a comparative analysis of the *ves* gene repertoire in *Babesia*, we have produced high-quality, draft genome sequences for an additional strain of *Babesia bovis* (C9.1), four strains of *B. bigemina* (BOND, PR, BbiS3P and JG29) and two strains of *B. divergens* (1802A and Rouen1987). Descriptions of these strains and genome sequences are given in Table 1. Variation in genome size, gene number and the proportion of coding sequences are related to the quality of sequence assemblies and sequence contiguity, which is lower in sequences produced from short reads only. The larger number of coding sequences in *B. bigemina* relative to other species is due to unique gene duplications of conserved gene families and to a greater number of species-specific sequences encoding hypothetical proteins. Nonetheless, clustering analysis of *B. bovis* T2Bo coding sequences combined with corresponding data from *B. bigemina* BOND and *B. divergens* 1802A shows that gene content is consistent between species and that variation in surface antigens occurs against a largely conserved genomic background. Figure 1 shows that coding sequences seen in all species represent 78–88% of genes; 68–81% of all coding sequences show one-to-one correspondence (i.e. perfect orthology). Therefore, species-specific genes represent between 12.2 and 21.5% of genes in these species, with *B. bigemina* displaying the highest proportion of unique features. These are maximum estimates since they include predicted protein sequences that failed to cluster, and so potential mis-annotated sequences could be designated as species-specific genes. *Ves* gene homologs comprise a large proportion of these species-specific genes: 4.7% of all coding sequences in *B. bovis* T2Bo, 8% in *B. bigemina* BOND and 11.2% in *B. divergens* 1802A. While the remaining unique sequences are not homologous to *ves1*, they are often predicted to be expressed at the cell surface. This prediction is based upon their overall structural similarities with the *B. bovis* VESA1 polypeptides, which are placed on the erythrocyte surface (42), including a well-conserved C-terminal end and predicted transmembrane domain. This indicates that the most dynamic features of these genomes are associated with the host-pathogen interface.

**Table 1.** Properties of seven *Babesia* genome sequences produced in this study

			Genome sequence					Genome content				
Strain	Origin	Host	Size (Mbp)	pGC	No. of scaffolds	N50 (Mb)	Mean coverage	No. of genes	% Coding	Mean gene length (bp)	% Genes with introns	Gene density (bp)
*B. bovis* C9.1	Mexico	Cow	7.61	42	46	2.05	591	3726	70.5	1501	62.53	2154
*B. bigemina* BOND	Argentina	Cow	13.84	51	6	3.52	8	4457	66.3	1531	58.44	2306
*B. bigemina* PR	Puerto Rico	Cow	12.68	50	320	2.46	362	4723	NA	1812	59.26	ND
*B. bigemina* BbiS3P	Argentina	Cow	12.94	50	533	3.0	218	4948	NA	1805	58.59	ND
*B. bigemina* JG29	Mexico	Cow	15.9	50	1299	2.11	262	5689	NA	1752	54.23	ND
*B. divergens* 1802A	France	Cow	9.58	42	81	1.12	43	4134	64.0	1487	59.02	2321
*B. divergens* Rouen1987	France	Human	8.97	46	482	NA*	736	4097	NA	1439	57.51	ND

Note. All statistics refer to contigs greater than 1 kb in size. Due to the number of sequencing gaps, entries marked ‘ND’ could not be calculated. Entries marked ‘NA’ are omitted because the contigs were ordered against an arbitrary union file of all contigs.

### *Ves*-like loci coincide with chromosomal breakpoints

**Figure 2.**
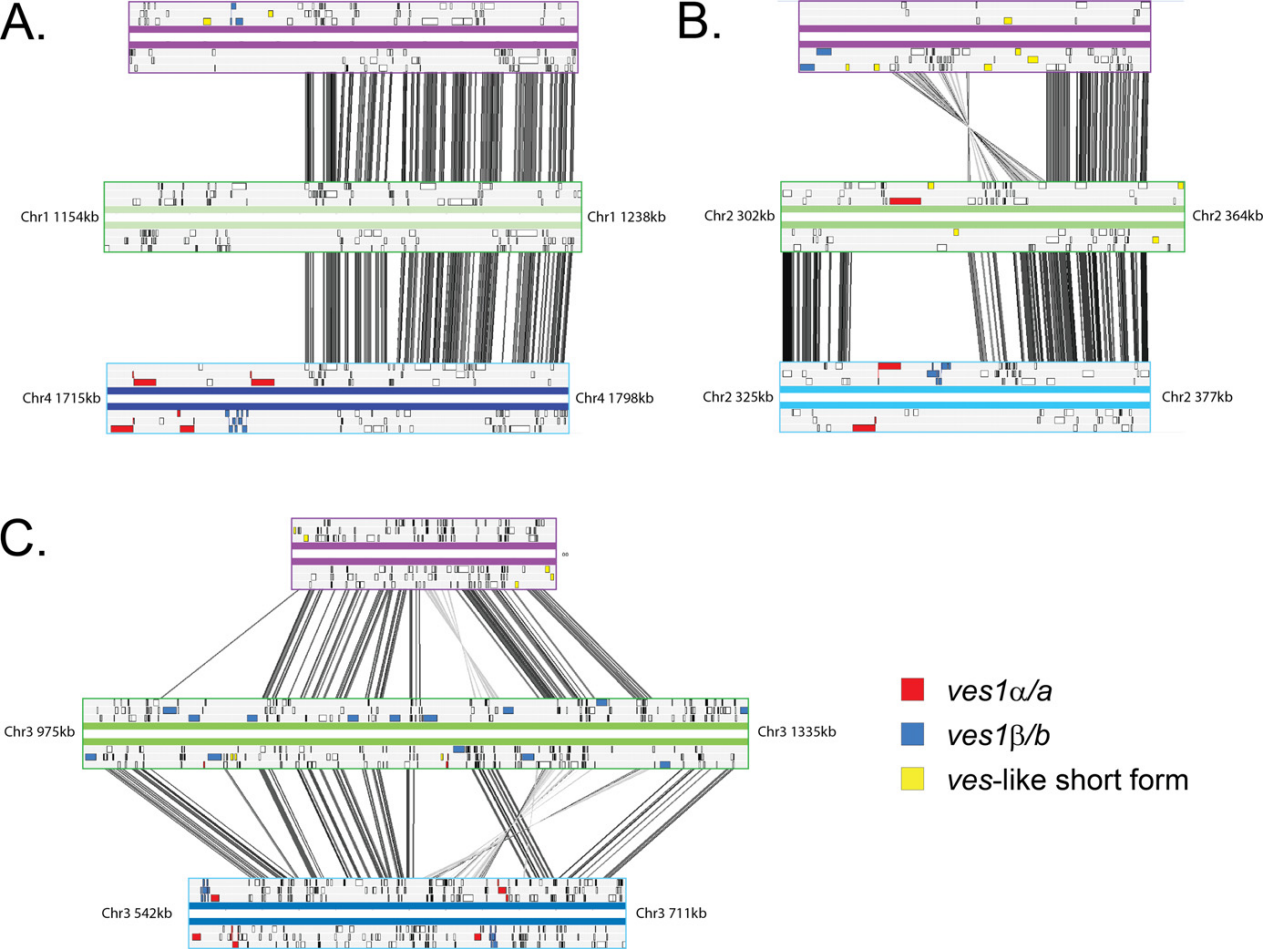
Comparison of gene order at three regions of chromosomal rearrangement. Forward and reverse strand are represented by horizontal bars, colour-coded by species (purple: *Babesia divergens* 1802A; green: Babesia *bigemina* BOND; blue: Babesia *bovis* T2Bo). Genes are indicated by boxes within reading frames. *ves*-like gene models are colour-coded as indicated by the key. Vertical grey bars between genomes represent significant BLASTn hits as calculated in ACT. (**A**) The region spanning 1154–1238kb of chromosome 1 in *B. bigemina*, which corresponds to chromosomal breakpoints in both other species. (**B**) The region spanning 302–364 kb of chromosome 2 in *B. bigemina*, which corresponds to a chromosomal breakpoint in *B. divergens*. (**C**) The region spanning 975–1335 kb of chromosome 3 in *B. bigemina* that is conserved in both other species but which has experienced numerous *B. bigemina*-specific insertions of *BbigVes1b* genes (shaded blue). The genomic locations of regions a-c are shown in Supplementary Figure S1A/B.

By mapping sequence scaffolds to the *B. bovis* genome, we determined that *B. bigemina* BOND has four chromosomes, as suggested previously (70); however, these are not co-linear with *B. bovis* chromosomes. Sequence alignment based on conserved gene order demonstrates that segmental inversion and chromosomal rearrangement have been very frequent since these *Babesia* species separated. Supplementary Figure S1A/B aligns various sequence blocks from the *B. bovis* T2Bo and *B. divergens* 1802A genomes that correspond to full-length chromosomes in *B. bigemina* BOND. The positions of coding sequences homologous to *B. bovis ves1* genes are marked and these coincide with chromosomal breakpoints. When we inspect breakpoints in co-linearity more closely, as shown in Figure 2, *ves*-like genes occur at homologous positions in all three genomes, but it is clear that these homologs have quite different size and orientation (Figure 2a). Other multi-copy genes (shaded yellow) also congregate at similar positions (Figure 2b), reminiscent of the proximity of *smORF* to *ves1* in *B. bovis* T2Bo (22). In addition, and somewhat anomalously, *B. bigemina* BOND has 28 *ves1b* loci on chromosome 3 at positions not associated with genome rearrangements, which likely represent lineage-specific insertion events (Figure 2c). The presence of *ves*-like genes at corresponding positions in different *Babesia* species may be ancestral, (i.e. these genes are orthologs, descended directly from a progenitor at the same position in the ancestral genome), or it may be mechanistic, (i.e. these positions are prone to rearrangement in all species and *ves*-like genes, which are not orthologous, independently transpose to them as regions in which purifying selection is weak).

### *Ves*-like sequences diverge rapidly after speciation

**Figure 3.**
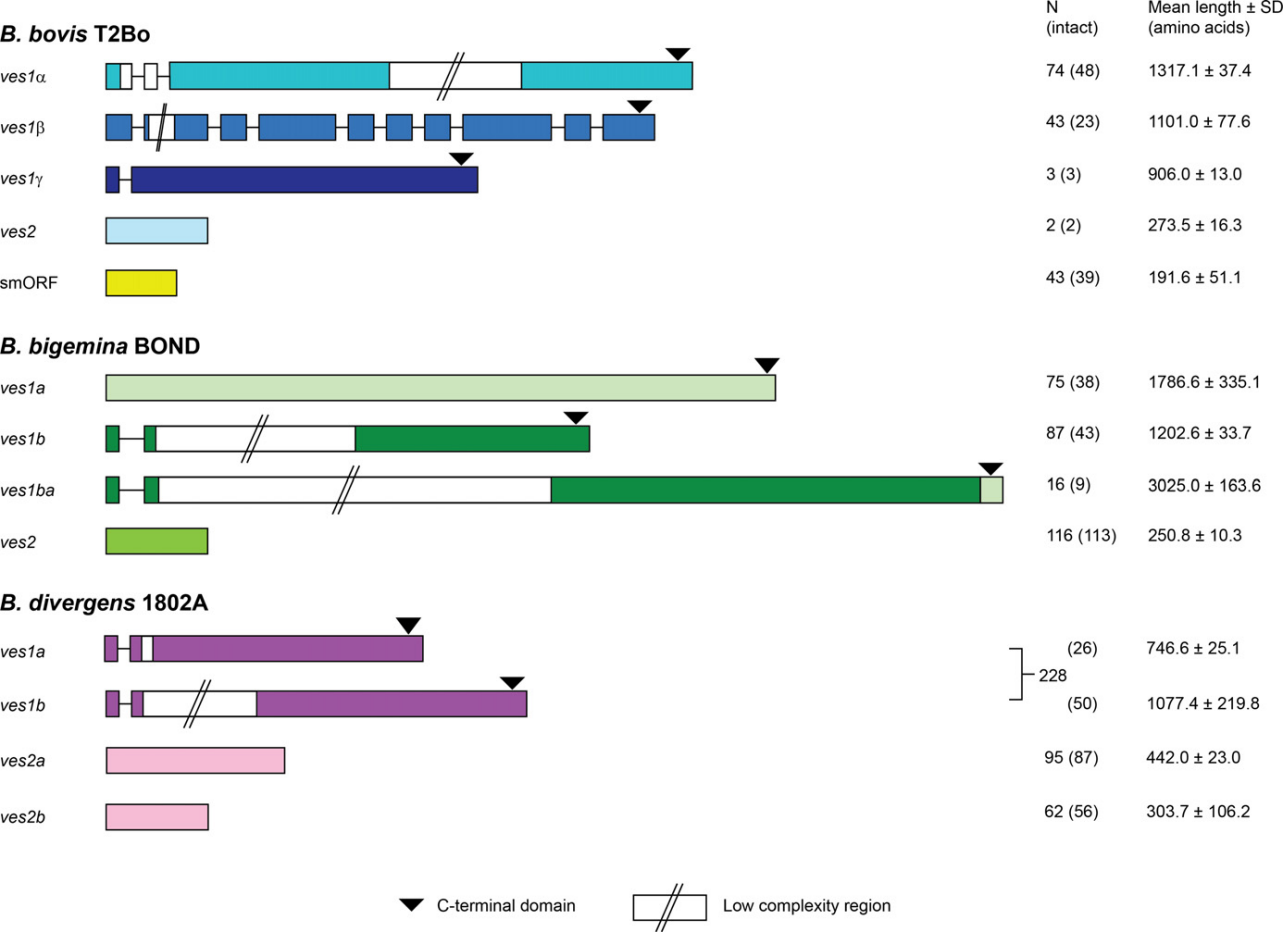
*Ves* gene repertoire in *Babesia* genome sequences. Gene models are drawn to scale (average lengths are shown) and are represented by shaded boxes (exons) and lines (introns). The presence of low complexity regions (typically repetitive and with variable length) and the conserved C-terminal domain (containing a single transmembrane helix) are indicated.

*Ves1* genes are among the fastest-evolving genes in *Babesia* genomes; in comparisons between species, they display about 25% amino acid identity and 75% of *ves1* genes occur in the top 25% of all genes when ranked by sequence divergence, which accounts for the skew towards low identity in frequency distributions of sequence divergence across the whole genome (see Supplementary Figure S2). Rapid divergence of VESA proteins is also evident in comparisons of genomic repertoire. The *B. bovis* T2Bo genome contains two families of *ves1* genes (*ves1α* and *ves1β*), as well as the *smORF* gene family that are found interspersed among *ves1* clusters. Through comparison with other genome sequences using BLAST and HMMER, we have identified *ves*-like genes in both *B. bigemina* BOND and *B. divergens* 1802A, although with important differences, as shown in Figure 3. Hereafter, we will prefix gene names with species labels, e.g. *BbovVes1α*, *BbigVes1a*, *BdivVes2a*, etc. While the relatively conserved C-terminal domain unambiguously confirms the homology of all *ves1* predicted proteins, these proteins are highly divergent in their remaining primary structures and no *B. bigemina* or *B. divergens* sequences contained extracellular ‘cysteine- and lysine-rich domain’ (CKRD) or the ‘variant domain conserved sequences’ (VDCS) domain previously characterized in *B. bovis* VESA (20).

**Figure 4.**
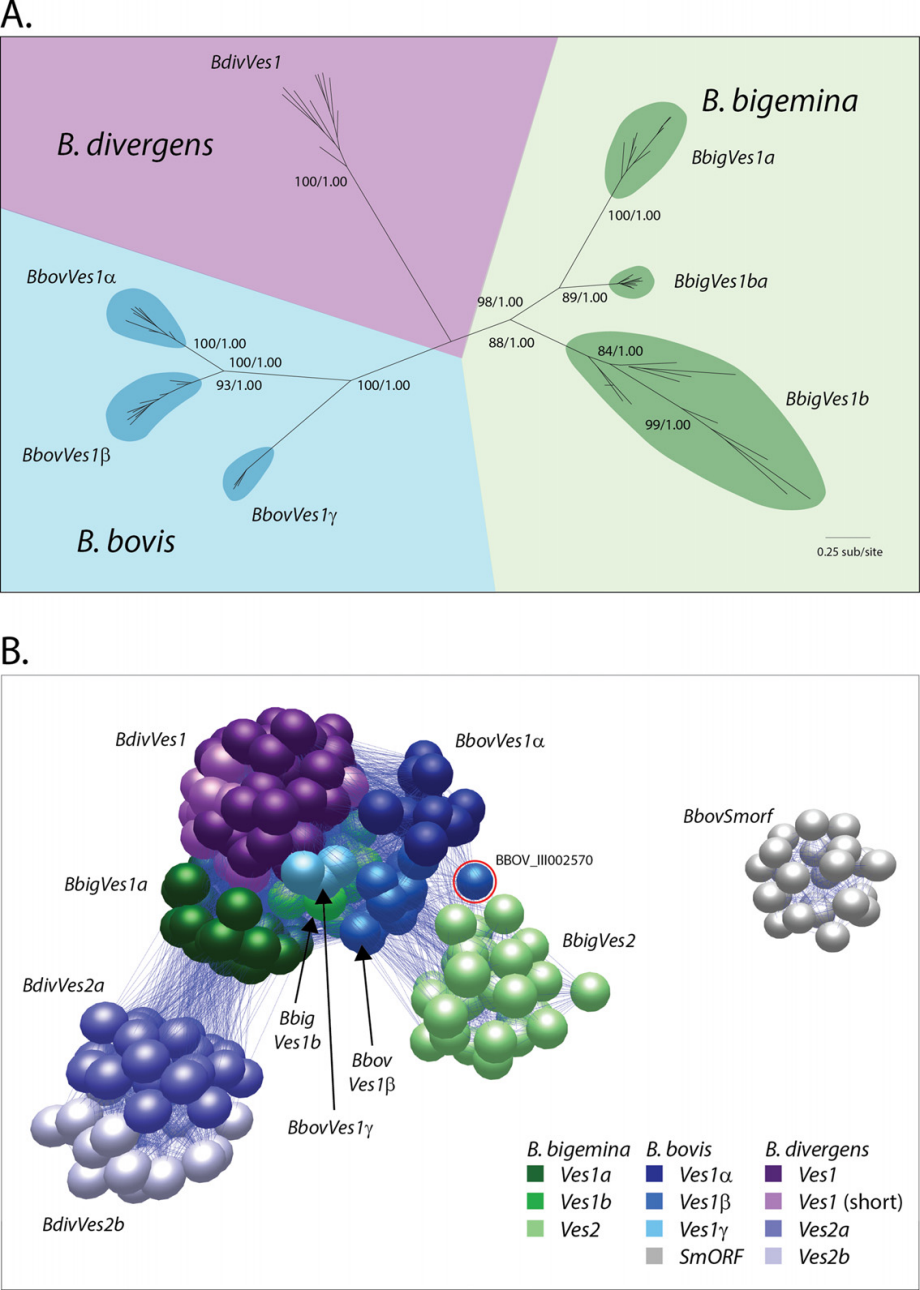
(**A**) Unrooted maximum likelihood phylogeny of *ves1* genes from *Babesia* spp. based on a multiple nucleotide sequence alignment corresponding to the conserved C-terminal domain of VESA1 only (840 characters). A GTR+Γ model was applied. Support for principal nodes is indicated by non-parametric bootstraps and posterior probabilities from a Bayesian analysis using the same model. (**B**) Sequence similarity network based on FASTA scores generated from pair-wise comparisons of VESA1 and VESA2 amino acid sequences and generated using BioLayout Express v3.0. Individual sequences are represented by spheres, shaded by gene family, connected by lines that represent sequence homology. The network was organized such that spheres edge length is minimized and spheres are positioned nearest to their closest relatives. A lower threshold has been applied to exclude poor sequence matches, leaving only the strongest similarities as determined by FASTA. SMORF sequences were included, but no FASTA scores exceeded the threshold. *Ves*-like gene families are labelled as described in the text; a single *Babesia bovis* sequence that clusters close to *BbigVes2* (BBOV_III002580) is shown with a red circle.

In *B. bigemina* BOND there are two families homologous to *ves1* that are approximately equally abundant; we refer to these families as *BbigVes1a* (*N* = 74) and *BbigVes1b* (*N* = 80). While ∼70% of *B. bovis ves1* are arranged in putative LATs consisting of both *ves1* types (26), these homologs are similarly arranged in only 35% of cases in *B. bigemina* BOND. Indeed, the phylogeny of all *ves1* genes (see Figure 4 below) indicates that these homologs in *B. bigemina* are not orthologous to the *ves1α* and *ves1β* of *B. bovis*. Sixteen gene copies (‘*BbigVes1ba’*) appear to be recombinant *ves1b*, with *ves1a*-type 3′ ends. We found no *smORF* homologs in *B. bigemina*.

In addition to *BbigVes1a* and *BbigVes1b*, there is a third gene family in *B. bigemina* that we refer to as *BbigVes2* (*N* = 116). These genes were identified due to their homology with the 5′-most 500 bp of *BbigVes1b* and they encode VESA-like proteins lacking the C-terminal domain of the canonical VESA1 or any GPI-anchor signal. These predicted proteins do not possess a PEXEL motif, which is required by some proteins in *Plasmodium* for secretion (73), but they do have a predicted signal peptide and, without any obvious means of membrane attachment, we suggest that they are secreted. *ves2* genes are located in similar positions to *ves1* in the *B. bigemina* genome (see Supplementary Figure S1). Comparison of the homologous region shared by *ves1b* and *ves2* (not shown) shows that all *ves2* gene copies share a common ancestor and are structurally distinct from all *ves1b* copies, demonstrating the *ves2* are not pseudogenic fragments of *ves1*.

In *B. divergens* 1802A, we observe a single gene family homologous to *ves1* (*N* = 202) that encode predicted proteins with a canonical VESA C-terminal domains for membrane attachment. This family includes two forms shown in Figure 3, one short and another longer due to a cysteine-rich, low complexity region towards the N-terminus that is absent from the short forms; otherwise these two forms are closely related and do not form monophyletic groups in phylogenetic trees (i.e. long and short forms are paraphyletic; see Figure 4). These genes are arranged in divergent orientation, suggestive of an LAT, in only three instances, although the abundance of gaps in the *B. divergens* assembly substantially limits our ability to quantify genomic context. The impression is, however, that *ves*-like genes in *B. divergens* are typically arranged in tandem at sub-telomeric loci, but rarely on opposing strands like the LAT. As with *B. bigemina*, we found no *smORF* homologs in *B. divergens* but we did identify *ves*-like genes that are homologous to the 5′ region of *ves1*, encoding predicted proteins without transmembrane attachment. In *B. divergens* these genes separate into two families [referred to as *BdivVes2a* (*N* = 95) and *BdivVes2b* (*N* = 62)], both of which are reciprocally monophyletic with respect to *ves1*, and therefore represent distinct lineages rather than partial *ves1* pseudogenes (see Figure 4).

With the discovery of *ves2* in the *B. bigemina* and *B. divergens* genomes, it is intuitive to suggest that *ves2* are analogous to *smORF* in *B. bovis*. There is no compelling evidence for homology between *smORF* and *ves*-like genes, and so it seems most likely that *smORF* has evolved in *B. bovis* independently to perform a role analogous to *ves2*. We chose to include *smORF* in our subsequent analyses because, as a multi-copy family of secreted proteins, showing clonal variation (74) and spatial association with *ves1*, *smORF* clearly share similar circumstances to *BbigVes2* and *BdivVes2*. We checked the *B. bovis* genomes for genuine orthologs to *ves2* and discovered a tandem gene pair (BBOV_III002570/2580), which encode homologs to *BbigVes2* in both *B. bovis* strains. Hence, these genes may be the sole remaining representatives of a *BbovVes2* family now largely lost, perhaps replaced by *smORF*.

*Ves1* genes share a 3′ region that encodes the conserved VESA C-terminus. This is the only region for which a multiple sequence alignment can be made across all species. A maximum likelihood phylogeny was estimated from the 840-character sequence alignment using PHYML (see Figure 4a). The phylogeny indicates that, while the ancestor of these *Babesia* species possessed a *ves1* gene family, the dimorphism evident in *B. bovis* (*BbovVes1α/1β*) and *B. bigemina* (*BbigVes1a/1b*) is not ancestral and has independent origins in these lineages. Given the large genetic distances between these paralogous families within the same species, this indicates very substantial structural innovation post-speciation on multiple occasions. If we compare the branch lengths between *BbovVes1α/1β* (0.5 substitutions per site) and *BbigVes1a/1b* (1.5), the paralogs in *B. bovis* appear to have diverged much less. This is consistent with the hypothesis that *BbovVes1α* evolved from *BbovVes1β* after the origin of *B. bovis* through reverse transcription, i.e. integration of an incompletely spliced *BbovVes1β* transcript, which was originally proposed to account for the lack of introns in *BbovVes1α* (22) *.* Furthermore, we have identified a third *ves1* sequence type in the *B. bovis* T2Bo genome not previously reported (*BbovVes1γ*; *N* = 3). Figure 4a shows that the branch length between *BbovVes1γ* and *BbovVes1α/1β* (1.5) is consistent with the distance between paralogous families in *B. bigemina* (1.46), suggesting that *BbovVes1α* may have substituted *BbovVes1γ* as one half of the heterodimer.

The relationships of *ves2* are not shown in Figure 4a because *ves2* lack the conserved C-terminal region of the canonical forms. Therefore, we generated a network based on pair-wise FASTA scores (Figure 4b), exhaustively comparing full-length VESA1 and VESA2 sequences from all species, to examine the suggestion that *ves2* genes independently evolved from ancestral *ves1* sequences. Canonical VESA1 sequences cluster together at the centre of the network, while protein sequences corresponding to *BbigVes2* and *BdivVes2a/2b* share no connections. This is consistent with *ves1* in all species having a single common origin, as implied in Figure 4a, while *ves2* have separate origins. *BdivVes2a/2b* unambiguously cluster most closely to *BbigVes1a*, suggesting that they are derived from an ancestor of *BbigVes1a* now lost or unrecognizable in *B. divergens*. *BbigVes2* clusters most closely to *BbigVes1b* and *BbovVes1α*, suggesting that it was derived from an ancestor of these two gene families. The rare *BbovVes2* sequences introduced above are positioned intermediately between *BbigVes2* and *BbovVes1β* in Figure 4b (circled in red), suggesting that may share the same origin as *BbigVes2*. Therefore, given that a *ves1* structure is present in all species (and, indeed, in *B. microti* (17)), but a common *ves2* structure is not widespread, we may infer that *ves2* in *B. bigemina* and *B. divergens* are not orthologous, and that they have been derived from *ves1* (and not *vice versa*) on separate occasions.

### Multiple VESA2 proteins may be expressed with greater abundance than VESA1

**Figure 5.**
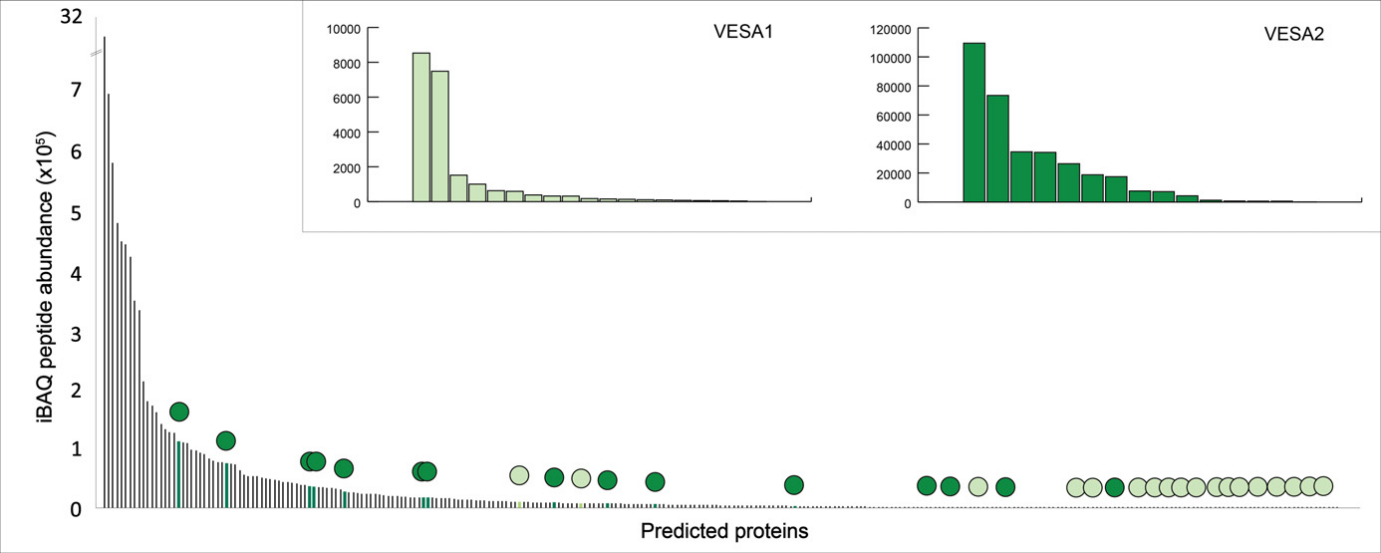
Frequency histogram showing ranked abundance of peptides detected in proteomic analysis of *Babesia bigemina* PR. The position of VESA1 (light green) and VESA2 (dark green) predicted proteins are shown with filled circles. Frequency histograms of VESA-like proteins only are shown in the insets.

Genome comparison indicates that *ves*-like genes lacking the conserved 3′ region have been independently derived from the canonical *ves1* genes during *Babesia* evolution. Sequence comparison indicates that these *ves2* genes are not non-functional *ves1* pseudogenes but, in fact, encode highly abundant and polymorphic proteins. To explore the biological differences between *ves1* and *ves2*, we carried out proteomic analyses of global gene expression in *B. bigemina* PR. We identified 1777 peptides corresponding to 366 predicted proteins in the *B. bigemina* BOND reference genome (i.e. 8.2% of the predicted proteome; see Supplementary Table S1). Figure 5 shows the abundance of VESA1 and VESA2 protein relative to each other, and to all other proteins identified in our data set. Most importantly, it shows that of the 15 different VESA2 proteins that are expressed, seven are more abundant than the most abundant VESA1 protein; in general VESA2 are significantly more abundant than VESA1 (*P* < 0.001; randomization test). The distribution of abundance between proteins is also subtly different. We observe two co-dominant VESA1B proteins (BBBOND_0303890 and BBBOND_0305310) and 17 other copies expressed at a low level (i.e. between 0.08 and 17.7% of the abundance of the dominant protein). This observation could be consistent with either the biallelic expression of a mixed VESA1B homodimer (we did not detect expression of VESA1A) or monoallelic expression of VESA1b monomers in the mixed population. The results also suggest that either multiple VESA2 are expressed simultaneously or there is rapid switching among *ves2* genes. *B. bigemina* PR is not a clonal line, and our data cannot distinguish these possibilities. In contrast to VESA1, the most abundant VESA2 protein (BBBOND_0302650) showed much less conspicuous dominance over other expressed VESA2 (see Figure 5). Hence, VESA2 expression may be less regulated than VESA1, although what we observed constitutes only 23% of the *ves2* repertoire in *B. bigemina* PR. These results confirm that multiple *ves2* genes are competent to be expressed and are not simply degenerate *ves1* gene copies.

The apparent lack of VESA1A expression is puzzling; it is unlikely to be due to technical insensitivity since we are able to detect a variety of VESA1B and VESA2 peptides at even very low abundance. To check this result, we carried out RNA-seq analysis of *ves*-like transcripts and this corroborated the proteomic data in showing little evidence for *ves1a* expression (Supplementary Figure S3). It is possible that, if expressed individually and monoallelically, there may be *in vitro* selection over many generations against parasites expressing VESA1A, e.g. by adhesiveness towards polystyrene, removing these parasites from the population. This observation deserves further investigation.

### Transposition of *ves* genes is evident from intraspecies variation

It is apparent from the species comparisons above that *ves1* and *ves2* genes are highly labile in terms of their molecular sequences and their genomic repertoire. To achieve such substantial divergence, we must predict that *ves* genes are frequently transposed to new genomic positions over ecological or individual timescales. To observe transposition directly we sequenced the genomes of additional strains of *B. bovis, B. bigemina* and *B. divergens* from diverse locations (see Table 1) and determined the conservation of *ves* loci at the intraspecific level. Table 2 describes the considerable variation within species in the presence and absence of *ves* loci. For example, between 65.3 and 69.4% of *BbigVes1a* loci and between 78.2 and 80.8% of *BbigVes2* loci are present in multiple *B. bigemina* strains. However, phylogenetic analysis of *ves* gene repertoires from multiple strains produced no evidence for strain-specific gene family expansions that might suggest structural differentiation of *ves* repertoires within a species (data not shown). Thus, while *ves* gene repertoire among divergent *B. bigemina* strains overlaps structurally and phylogenetically, a sizable minority of *ves1* genes are absent from conserved genomic positions in any given instance, indicating that gene transposition is much more frequent than either gene duplication or the evolution of novel sequences through amino acid substitution.

**Table 2.** Percentage values for presence, absence and orthology of *ves* loci in *Babesia* reference genome sequences

Reference	*Ves* gene family	*N*	Strain	% Loci			
				Present	No assembly	Absent	Orthologous
*B. bovis* T2Bo	*BbovVes1α*	82	C9.1	51.3	22.4	26.3	25
	*BbovVes1β*	50	C9.1	54.5	25	20.5	12.5
	*smORF*	43	C9.1	55.8	14	30.2	75
*B. bigemina* BOND	*BbigVes1a*	78	PR	65.3	6.7	28	23.5
			JG29	66.6	2.6	30.8	32.2
			BbiS3P	69.4	0	30.6	26
	*BbigVes1b*	79	PR	40	43.7	16.3	0
			JG29	46.6	37.5	15.9	1.8
			BbiS3P	37.5	42.5	20	3.3
	*BbigVes2*	116	PR	78.2	0	21.8	79.5
			JG29	80.8	0	19.2	81.3
			BbiS3P	80	2.6	17.4	85.1
*B. divergens* 1802A	*BdivVes1*	28	Rouen1987	84.6	-	15.3	59.1
	*BdivVes2*	113	Rouen1987	73.4	-	26.6	86.3

Note. When a comparison was not possible due to sequence gaps in the *B. bovis* and *B. bigemina* genomes (mostly affecting sub-telomeric regions), this was recorded as ‘No assembly’. This was not recorded for *B. divergens*, for which most *ves*-like genes are located on unscaffolded contigs, and percentage values for this species refer to only those loci that were confirmed in the same genomic context in both 1802A and Rouen1987 genome sequences.

Moreover, even when genes are present at the same locus in multiple strains, these are not always orthologous; indeed, Table 2 shows that the majority of *ves1* in *B. bovis* and *B. bigemina* are not orthologous when conserved in position. Conversely, 75% of conserved *smORF* loci contain orthologous sequences, and likewise 79.5–85.1% of *BbigVes2* and 86.3% of *BdivVes2* are orthologous. Supplementary Figure S4 illustrates this distinction for *B. bigemina ves1* and *ves2* loci across four strains, contrasting the complete conservation of a *ves2* locus with three *ves1* loci for which micro-homology has broken down to some extent. While the orthologous sequences at the *ves2* and upstream *ves1* loci cluster together in the global gene family phylogenies, sequences from the downstream *ves1* loci, which are not orthologous, cluster apart.

From the results above, widespread transposition of *ves* sequences is apparent. To assess its significance we adopted a co-phylogenetic approach, as described in Supplementary Figure S5. Taking pairs of strains in turn, we estimated maximum likelihood phylogenies for *ves1* and *ves2* genes that occurred at conserved positions, excluding *ves* genes at positions that were unique to a single strain. If transposition has not occurred, these phylogenies should be identical, or at least display variations not significantly greater than expected by systematic error, because the genes would be orthologous and diversified in the common ancestor of the two strains concerned. If strain phylogenies are significantly different, then this means that, despite being found at conserved positions, gene sequences have changed since the strains originated, which we interpret as evidence for transposition through gene conversion. It is likely that the individual genes had at one time been the active locus for *ves1α/a* and/or *ves1β/b* transcription (LAT). While the LAT, the genes would have undergone repetitive events of segmental gene conversion with formation of extensive mosaics, as previously described (22).

**Figure 6.**
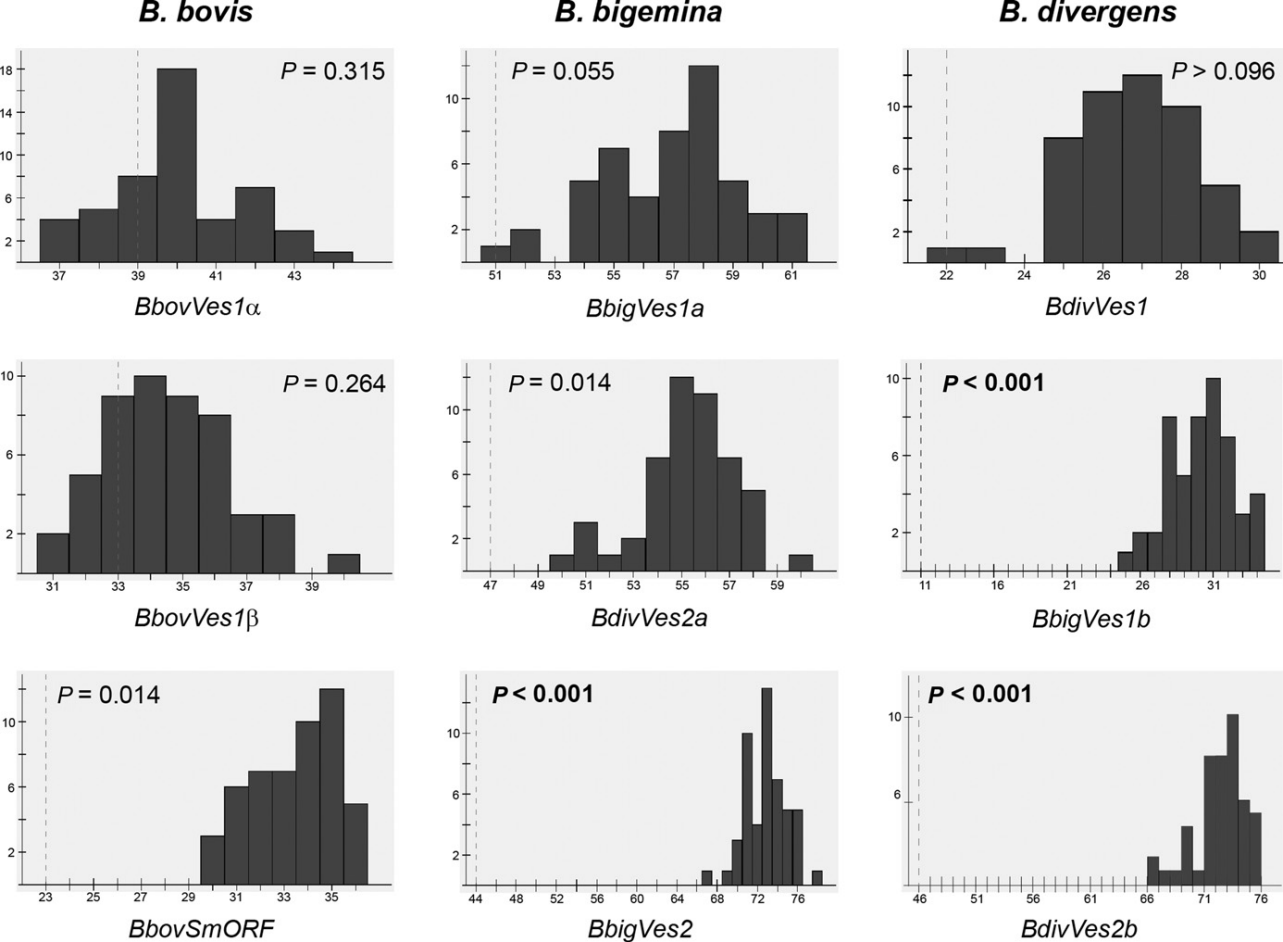
Comparison of event costs required to reconcile *ves1* and *ves2* gene phylogenies. For each *ves1* and *ves2* gene family, phylogenies were estimated for positionally-conserved genes, i.e. loci conserved in both the reference strain and one other strain. In the absence of recombination after the strains diverge, such trees should have the same topology. Significance of topological congruence is assessed through phylogenetic reconciliation using the programme Jane 4, whereby evolutionary events are posited to explain topological disparities between the trees. Each histogram shows the frequency distribution of event costs for 100 randomized trees generated by permuting the reference strain phylogeny, compared to the observed event cost (vertical dashed line). Where observed and randomized event costs overlap, this indicates that there is no significant agreement between the trees, which we interpret as evidence for recombination. *P*-values represent the probability of obtaining the observed cost in randomized co-phylogenies (i.e. of observed tree similarity being due to chance), and are mean averages taken over all cost combinations.

Congruence between strain phylogenies was assessed for each *ves*-like subfamily using permutation tests executed in the programme Jane 4 (69) and judged to be significant if *P* < 0.01. For each panel in Figure 6, the observed ‘cost’ of reconciling incongruence between strain phylogenies is marked by a vertical red line; adjacent to this is a frequency distribution showing the costs of reconciliation when the phylogenies are randomized. The distance between the observed and randomized costs is a reflection of the congruence in the trees, where they overlap congruence is likely to be significant. Contrary to the null expectation, Figure 6 shows that *ves1* phylogenies were incongruent, (i.e. shared a level of topological similarity no greater than that due to chance), for *B. bovis* (*BbovVes1a*, *P* = 0.315; *BbovVes1b*, *P* = 0.264), *B. bigemina* (*BbigVes1a*, *P* = 0.055; *BbigVes1b*, *P* = 0.014) and *B. divergens* (*P* = 0.1). In contrast, strain phylogenies for *BbigVes2*, *BdivVes2a* and *BdivVes2b* were significantly congruent (*P* < 0.001). Thus, when *ves1* genes are present at the same genomic position in different strains they almost invariably have different sequences, whereas when *ves2* genes are conserved in position, they are also often conserved in sequence (i.e. are orthologous).

### *ves1* and *ves2* genes differ in their exposure to recombination

**Figure 7.**
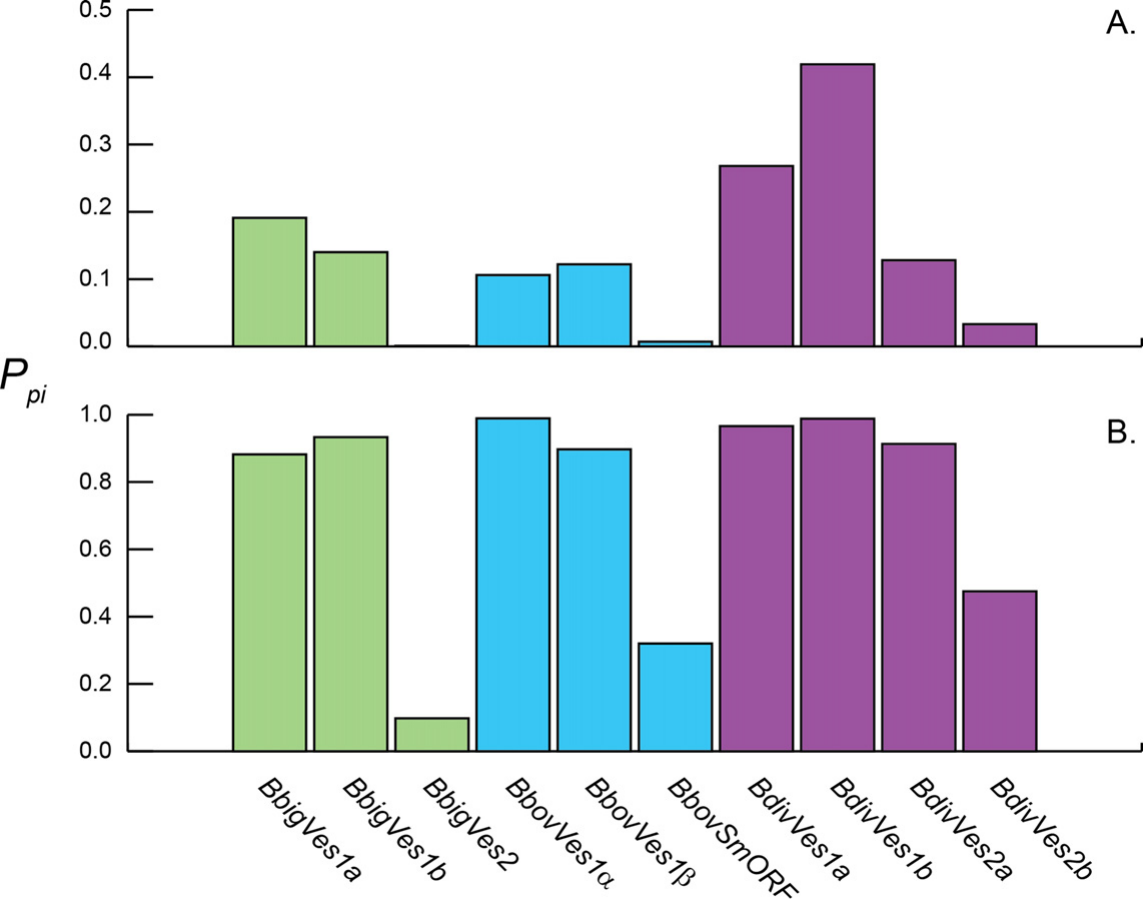
Evidence for recombination among gene copies for *ves*-like gene families in *Babesia* spp. using two programs: 3seq (**A**) and PhiPack (**B**). The proportion of sub-alignments showing significant phylogenetic incompatibility (*P_pi_*) is shown for *ves1* and *ves2* gene families, with bars shaded by species as previously.

The transposition of *ves1* and, to a lesser extent, *ves2* sequences between loci is consistent with the idea that ectopic gene conversion of *ves* sequences is frequent and important in driving antigenic variation (22). Sequence mosaicism has been observed in *BbovVes1* genes and is interpreted as evidence for historical recombination (22). We sought to compare the role of recombination between *ves1* in different species, and between *ves1* and *ves2* in the same genomes, by estimating the frequency of sequence mosaicism using phylogenetic methods. Evidence for recombination was identified in the form of phylogenetic incompatibility (PI) among characters, when sampling alignments of full-length, *ves*-like sequences. Two programs, PhiPack (70) and 3seq (71), were used to assess the significance of PI in multiple alignments sampled from all full-length sequences (see methods). The metric *P_pi_* records the proportion of alignments that returned significant PI (i.e. *P* < 0.05) for any given sub-family. As Figure 7 shows, *ves1* genes in all three species displayed significant PI. Using 3seq, *P_pi_* tended to be in the 0.1–0.25 range, although *BdivVes1b* sequences displayed PI much more frequently (*P_pi_* = 0.42). Using PhiPack, *P_pi_* was between 0.88–0.99 for the same gene families. The difference between methods likely results from the use of four sequences in PhiPack analyses (one more than in 3seq analyses), which increases the chances of observing PI. The increased *P_pi_* in *BdivVes1b* was not observed when using PhiPack.

**Figure 8.**
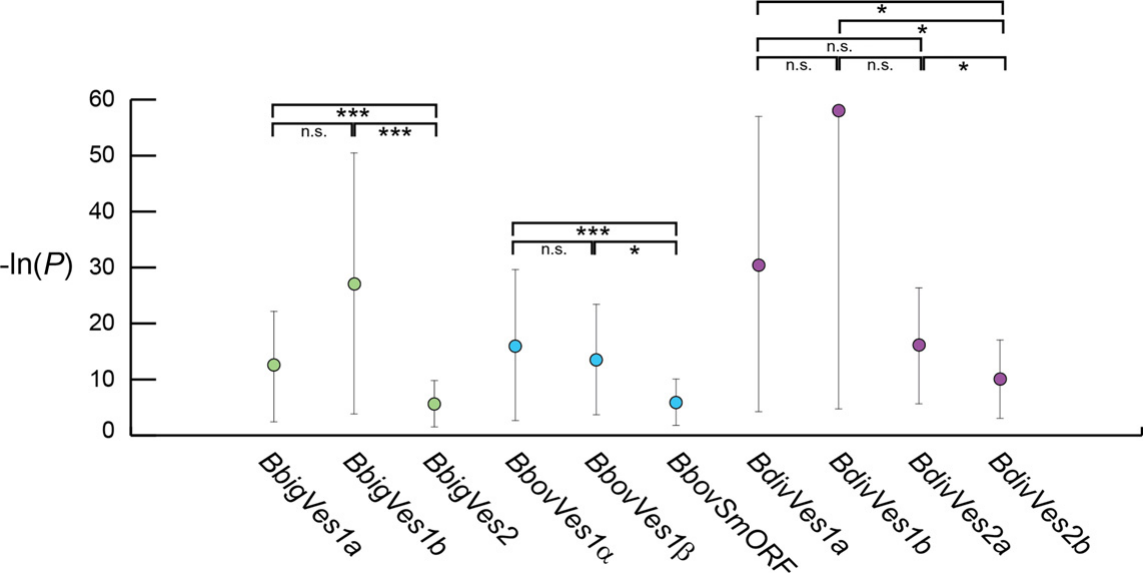
Comparison of *P*-values in tests for phylogenetic incompatibility (using 3seq). Mean *P*-value is shown for each *ves1* and *ves2* gene family ± one standard deviation. The values are converted to a negative natural log scale for ease of comparison. Gene families are shaded as previously. The results of pair-wise t-tests between all *P*-values returned in each analysis are indicated (n.s. = not significant). Note that most *P* values for *BdivVes1b* tests were zero, although a minority returned non-zero (but highly significant) log values. This circumstance is responsible for the apparently large standard deviation around this mean. In fact, all *BdivVes1b* sequence triplets returned highly significant tests for phylogenetic incompatibility. *** *P* < 0.001, * *P* < 0.05.

A clear difference in *P_pi_* is observed between *ves1* and *ves2* subfamilies using both programs. In co-phylogenetic analyses, *BbigVes2* and *BdivVes2b* showed little evidence of transposition or *in situ* gene conversion. This is corroborated in Figure 7, where *P_pi_* is 0.001 and 0.033 respectively using 3seq. *SmORF* and *BdivVes2a* co-phylogenies were more equivocal but these two subfamilies also offer less evidence for recombination than among *ves1* sequences (*P_pi_* = 0.007 and 0.128 respectively). When we compare the actual *P*-values associated with 3seq analyses of all sequence triplets (Figure 8), these are significantly lower for each *ves2*/*smORF* subfamily relative to *ves1* genes in the same genome. The exception here is *BdivVes2a*, which has a comparable *P_pi_* to *BdivVes1* but a lower mean *P*-value. In summary, those sub-families showing greater congruence in co-phylogeny analyses tend to show significantly less PI in analysis of sequence mosaics, and so *ves1* and *ves2* sub-families display a markedly different evolutionary dynamic that is consistent across species. Although the function(s) of these proteins are not yet known, they are unlikely to be exposed on the infected erythrocyte surface. Immune selection pressure may have been greatly reduced following their derivation from *ves1* genes as a result, and they therefore show greater co-phylogeny.

The position of recombination breakpoints within *ves*-like sequence alignments could indicate low-complexity structural motifs that facilitate recombination by promoting strand annealing between homologous sequences (32). The most likely 5′ and 3′ breakpoints for recombinant tracts detected by 3seq can be plotted onto the sequence alignments for each *ves*-like gene family (Supplementary Figure S6). While there is no consistent pattern between families and sometimes no preference is shown at all, it appears that some breakpoints may be associated with repetitive regions that are unique to each *ves* gene family. For example, in the case of *BbovVes1α*, the 5′ breakpoint between 800 and 900 bp is immediately preceded by sequences encoding an alanine-rich repeat region and another domain containing cysteine-based repeats. The 3′ breakpoint between 2700 and 2800 bp is immediately preceded by sequences encoding the largest glycine/lysine-rich repeat region in the molecule. Similarly, *BbigVes1a* has a dominant breakpoint between 1250 and 1500 bp that immediately precedes sequences encoding a low-complexity region dominated by serine, threonine and proline residues. However, such regions are common in these molecules and so these associations will require further validation.

## DISCUSSION

In common with other pathogens, the most labile features of *Babesia* genomes are those genes encoding surface antigens. *Ves1* genes, and the shortened forms derived from them, display the greatest divergence in species comparisons and the most polymorphism in strain comparisons. These genes are also found consistently in genomic regions otherwise depleted of coding sequences and prone to rearrangement. At each level of inspection, our analyses have emphasized that *ves1*-like sequences are transposed between locations: the variation in presence or absence of individual loci, the non-orthology of sequences occupying homologous positions in different species and strains, the incongruence of *ves1* co-phylogenies, and the abundant evidence for recombination between *ves1* sequences. In each respect, *ves2* and *smORF* sequences are different; they are more consistently conserved in position, their co-phylogenies have greater congruence and their sequence alignments present significantly less evidence for sequence mosaicism.

Therefore, there is a fundamental difference in evolutionary dynamic between the canonical *ves1*, the evolution of which is perhaps determined by its particular requirement for transposition, and *ves2*, which evolves in a manner more consistent with a basic birth-death model, albeit with substantially more gene duplication than most gene families.

The genomic basis for immune evasion in *B. bovis* (19,20,22) is, in several respects, analogous to the expression of PfEMP1 from *var* genes in the malaria parasite *Plasmodium falciparum* (2), to the Variant Surface Glycoprotein (*vsg*) in the African trypanosome *Trypanosoma brucei* (75); and to the Major Surface Glycoproteins (*msg*) in the ascomycete *Pneumocystis carinii* (76). The convergent evolution of immune evasion functions among these proteins and their regulatory milieux in diverse organisms has been recognized (77–80) and perhaps this reflects their shared experience of a relatively conservative vertebrate immune response. Typically, an abundant gene family encoding a repetitive major surface protein is sited in labile regions of the genome; we have seen how *ves*-like genes are typically distributed at the most dynamic sites in the *Babesia* genome. Expression of the surface antigen is commonly restricted to a specific locus, while most or all other gene copies are silenced. Available evidence suggests this is the case for *ves1* genes in *B. bovis* (25) and perhaps *ves* genes among *Babesia* spp. in general (this study). Periodic switching of a new variant antigen gene, in whole or in part, into the expression site from silent loci, or epigenetic transcriptional switching among members of the multigene family, then results in antigenic variation on the parasite (or IRBC) surface.

The common role of gene conversion in antigenic variability across diverse organisms has also been highlighted (32,34,81). By detecting the signature of past recombination in multiple sequence alignments, recombination has been identified as a principal evolutionary force affecting gene family structure of *var* genes in *P. falciparum* (82–84), *vsg* in *T. brucei* (30,31,85) and *msg* in *P. carinii* (86). However, recombination of *var* genes appears to be meitoic, and gene conversion is not a demonstrated mechanism during antigenic variation within an individual host. Repetition within genes or in non-coding flanking regions is thought to facilitate recombination, suggesting that genome structure is adapted for increasing the mutability of variant antigen genes (4,87). Analogous motifs may exist in *Babesia*; the well-conserved C-terminal domains of *ves1* genes could plausibly act as annealing points during cross-over, while our recombination analyses have located breakpoints to repetitive parts of *ves1* genes. If so, they are unique to a given species, since they fall within regions that are not widely conserved among species.

What is recognized less is that these contingency gene families have similar phylogenetic patterns, i.e. variant antigen gene repertoires in related species do not overlap. There is no orthology between the *ves1* in different *Babesia* spp. (Figure 4), just as there are no orthologous *vsg* in different African trypanosome genomes (31,88), orthologous *var* in comparisons of *P. falciparum* and *P. reichenowi* (35), or indeed among *P. falciparum* strains (82). The mutual exclusivity of repertoires in these situations indicates rapid gene turnover; the frequent gain and loss of gene copies after speciation, resulting in the substitution of shared, ancestral characters by unique, derived ones. Hence, the rapid loss of phylogenetic diversity is a sampling effect; after speciation, species-specific sequence types are constantly created while ancestral types are a limited number, but all can be ‘overwritten’ by gene conversion. This likely explains the lack of cysteine-rich CKRD and VDCS domains encoded by *B. bovis ves* genes in those of *B. bigemina* and *B. divergens*. Moreover, a lack of these domains may explain the lack of cytoadhesive behaviour in *B. bigemina* and *B. divergens*, whereas *B. bovis* uses VESA1 as a highly variable cytoadhesion ligand (89). In addition to immune evasion, antigenic variation of VESA1 could provide for stochastic sampling of a very large molecular space on the endothelial cell surface for a complementary receptor.

Another feature of the rapid turnover of *ves*-like structures after speciation is the evolution of *ves2* from the N-terminal regions of *ves1*, which we believe has occurred on independent occasions. Although the functions of *ves2* and *smORF* are open questions, there are good reasons for believing that they are distinct from *ves1*. First, we have shown that *ves1* and *ves2/smORF* behave differently over evolutionary timescales. Second, they have radically different protein structures, and whereas VESA1 is membrane-bound in *B. bovis* (42) and we presume in *B. bigemina* and *B. divergens*, VESA2 and SMORF are strongly predicted to be secreted. Third, the two sequence classes have different expression profiles in *B. bigemina* PR, suggesting the employment of unlinked and perhaps different regulatory strategies.

We can speculate on *ves2* function through comparison with related Apicomplexan parasites. In *Plasmodium* spp., secretion is thought to be instrumental in remodeling host erythrocytes after the parasite has invaded. Altering erythrocyte structure is necessary for parasite metabolism and key survival strategies such as sequestration and antigenic variation (90–93). The secreted proteomes of *Plasmodium* spp. contain not only key effectors, for example, the PfEMP-1 protein in *P. falciparum*, but also diverse chaperones and cofactors thought to facilitate the expression of key effectors (94,95). So, we can imagine VESA2 being secreted into the cytoplasm of the IRBC as part of cocktail of parasite proteins that alter host cell structure. This might be in support of VESA1 expression in the IRBC plasma membrane directly, or to antagonize host proteins with the effect of making the IRBC more amenable to parasite survival, as happens when *Theileria* spp., a sister genus to *Babesia*, infect white blood cells (96–97). Alternatively, or perhaps additionally, since VESA2 proteins are expressed abundantly, they may serve to obfuscate the immune response. This could occur by creating a ‘smokescreen’ effect, inducing focus of dendritic cells upon antigens that are inaccessible and largely insensitive targets, analogous to the secretion of subtelomere-associated variant surface proteins (SVSP) into the host cytoplasm by *Theileria* spp. It has been suggested that presentation of SVSP peptides by class 1 MHC distracts the immune response from exposed antigens on the parasite surface (97) and, similarly, VESA2 may promote antibodies that have no reactivity to VESA1 (although without presentation of MHC, since *Babesia* spp. only infect erythrocytes). It remains to be seen if VESA2 are immunogenic, and if antibodies to VESA1 and VESA2 are cross-reactive.

In this study, we have confirmed that the genomic basis for antigenic variation in *B. bovis* is conserved throughout the genus, but with substantial, species-specific divergence in protein structure and gene repertoire. In fact, while *Babesia* genomes are broadly conserved in other respects, the evolution of *ves1* genes is a record of constant change and rapid turnover, which we suggest reflects the enduring challenge of host–parasite interactions over millions of years. In addition, *ves*-like genes with shortened predicted proteins have evolved from canonical *ves1* on independent occasions. These have distinct expression profiles (in *B. bigemina* at least) and evolutionary dynamics, suggesting a distinct function, though probably still involved in antigenic variation, or at the host–parasite interface. From previous observations of the *B. bovis* genome, it is thought that recombination between *ves1* gene copies could be crucial both to the mechanism of antigenic variation during infection, and to the generation of antigenic diversity in parasite populations (22,26). These results confirm the principal role of recombination across the genus and support the emerging view that genomic architecture facilitates recombination to both promote switching and generates antigenic diversity (98). As with *var* and *vsg*, the roles of recombination, rapid gene turnover and structural innovation in the evolution of variant antigens is affirmed in *ves1* phylogeny. The association of this phylogenetic pattern with variant antigen function in diverse parasite genomes is testament to the convergence in both structure and mechanism by diverse pathogens to combat vertebrate immunity.

## ACCESSION NUMBERS

Sequence read data have submitted to the European Nucleotide Archive (http://www.ebi.ac.uk/ena) with the accession numbers ERP000167 and ERP000252. BioProject identifiers (http://www.ncbi.nlm.nih.gov/bioproject/) for the *B. bigemina* BOND and *B. divergens* 1802A reference genomes are PRJEB5046 and PRJNA230984 respectively. Genome sequences for *B. bigemina* strains JG29, BbiS3P and PR, *B. bovis* C9.1 and *B. divergens* Rouen1987 are available from the Wellcome Trust Sanger Institute FTP site (ftp://ftp.sanger.ac.uk/pub/pathogens/Babesia/). Mass spectrometry and proteomic data have been deposited in the ProteomeXchange Consortium (http://proteomecentral.proteomexchange.org) via the PRIDE partner repository (http://www.ebi.ac.uk/pride/) with the dataset identifier PXD000629.

## SUPPLEMENTARY DATA

Supplementary Data are available at NAR Online.

SUPPLEMENTARY DATA
