# Supplementary material for: The evolutionary dynamics of variant antigen genes in Babesia reveal a history of genomic innovation underlying host–parasite interaction
Source: Nucleic Acids Res. 2014 May 5;42(11):7113–31. doi: 10.1093/nar/gku322 (PMC4066756; doi:10.1093/nar/gku322)
Supplement: SUPPLEMENTARY DATA [file supp_42_11_7113__index.html]

The evolutionary dynamics of variant antigen genes in Babesia reveal a history of genomic innovation underlying host–parasite interaction — SUPPLEMENTARY DATA 

# The evolutionary dynamics of variant antigen genes in *Babesia* reveal a history of genomic innovation underlying host–parasite interaction

## SUPPLEMENTARY DATA

**Files in this Data Supplement:**

- SUPPLEMENTARY DATA
- SUPPLEMENTARY DATA
