# Supplementary material for: The evolutionary dynamics of variant antigen genes in Babesia reveal a history of genomic innovation underlying host–parasite interaction
Source: Nucleic Acids Res. 2014 May 5;42(11):7113–31. doi: 10.1093/nar/gku322 (PMC4066756; doi:10.1093/nar/gku322)
Supplement: SUPPLEMENTARY DATA [file supp_gku322_nar-00013-z-2014-File010.docx]

**SUPPLEMENTARY DATA**

**
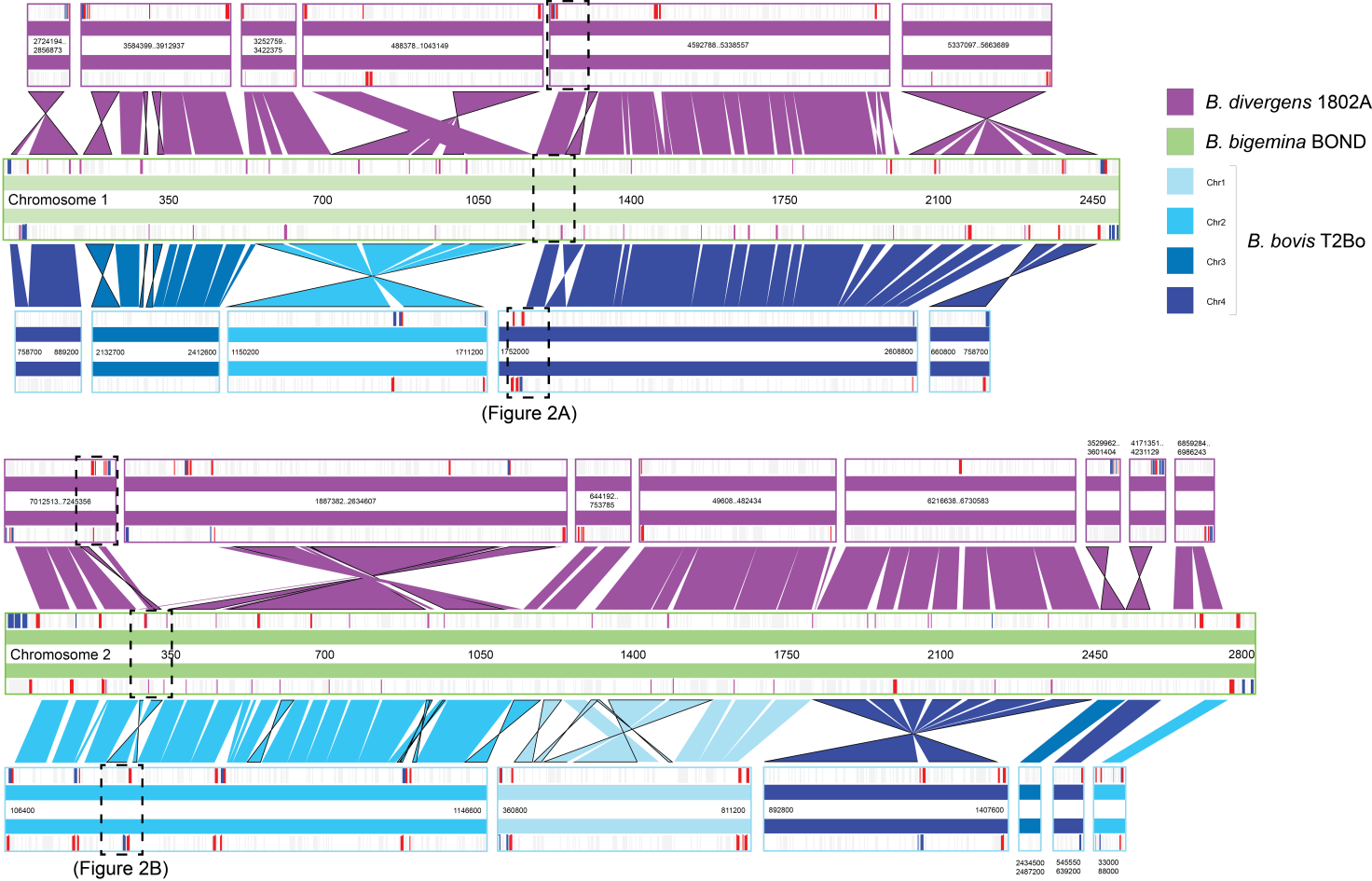
**

**Figure S1A**. **Comparison of gene order across chromosomes 1 and 2 in three *Babesia* species**. Chromosomes or sequence blocks with co-linear gene order are represented by horizontal, parallel bars and colour-coded by species. *B. bovis* sequence blocks are further colour-coded by chromosome number, while *B. divergens* blocks are not because chromosome identity is not known. Scale in base-pairs. Gene loci are represented by grey marks above each chromosome; *ves1α/a* and *ves1β/b* gene loci are represented by red and blue marks respectively. Vertical bars between chromosomes represent regions of conserved gene order, identified from significant tBLASTx matches. Areas bound by dashed lines are expanded in Figure 2a-b.


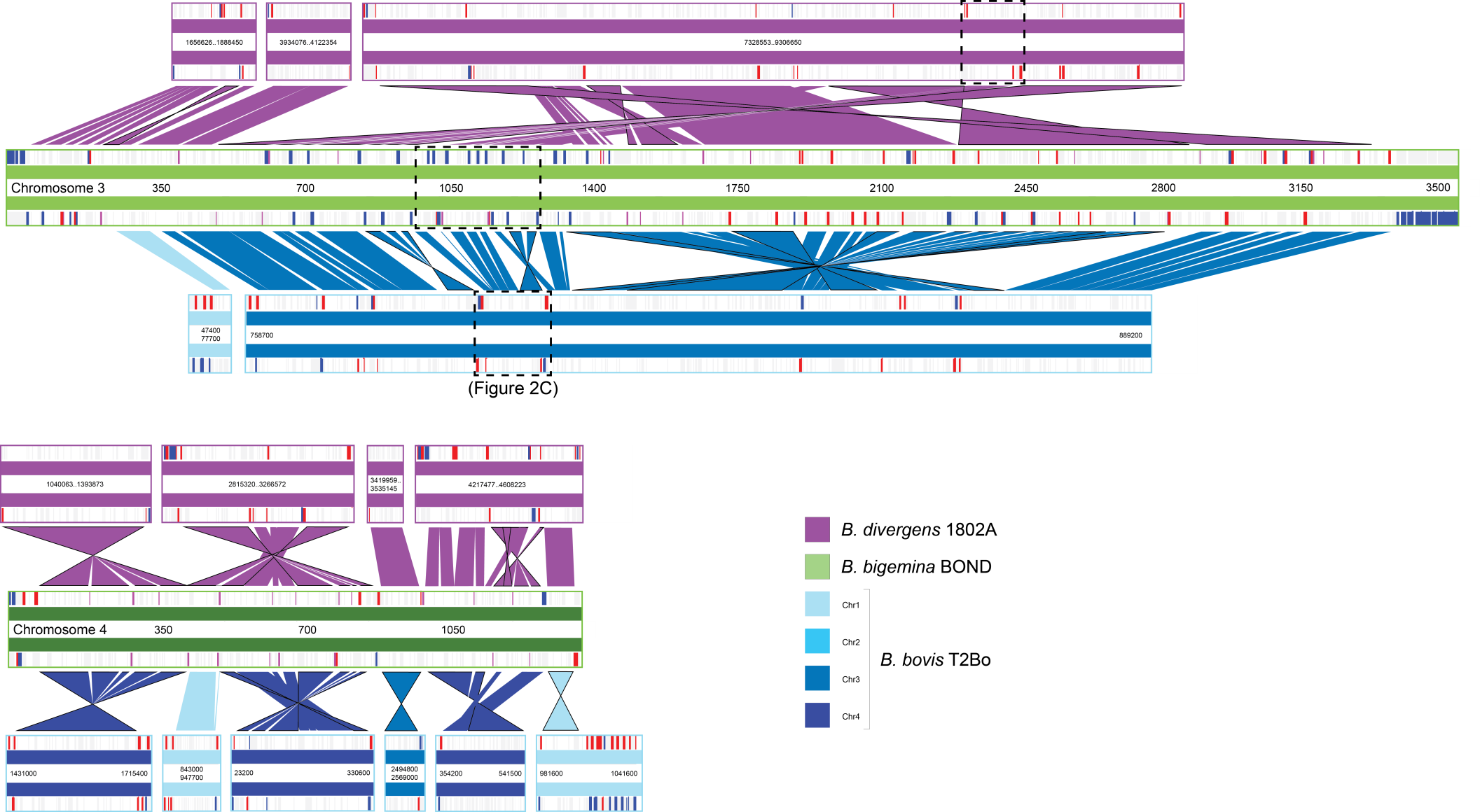


**Figure S1B**. **Comparison of gene order across chromosomes 3 and 4 in three *Babesia* species**. Chromosomes or sequence blocks with co-linear gene order are represented by horizontal, parallel bars and colour-coded by species. *B. bovis* sequence blocks are further colour-coded by chromosome number, while *B. divergens* blocks are not because chromosome identity is not known. Scale in base-pairs. Gene loci are represented by grey marks above each chromosome; *ves1α/a* and *ves1β/b* gene loci are represented by red and blue marks respectively. Vertical bars between chromosomes represent regions of conserved gene order, identified from significant tBLASTx matches. Areas bound by dashed lines are expanded in Figure 2c.

**
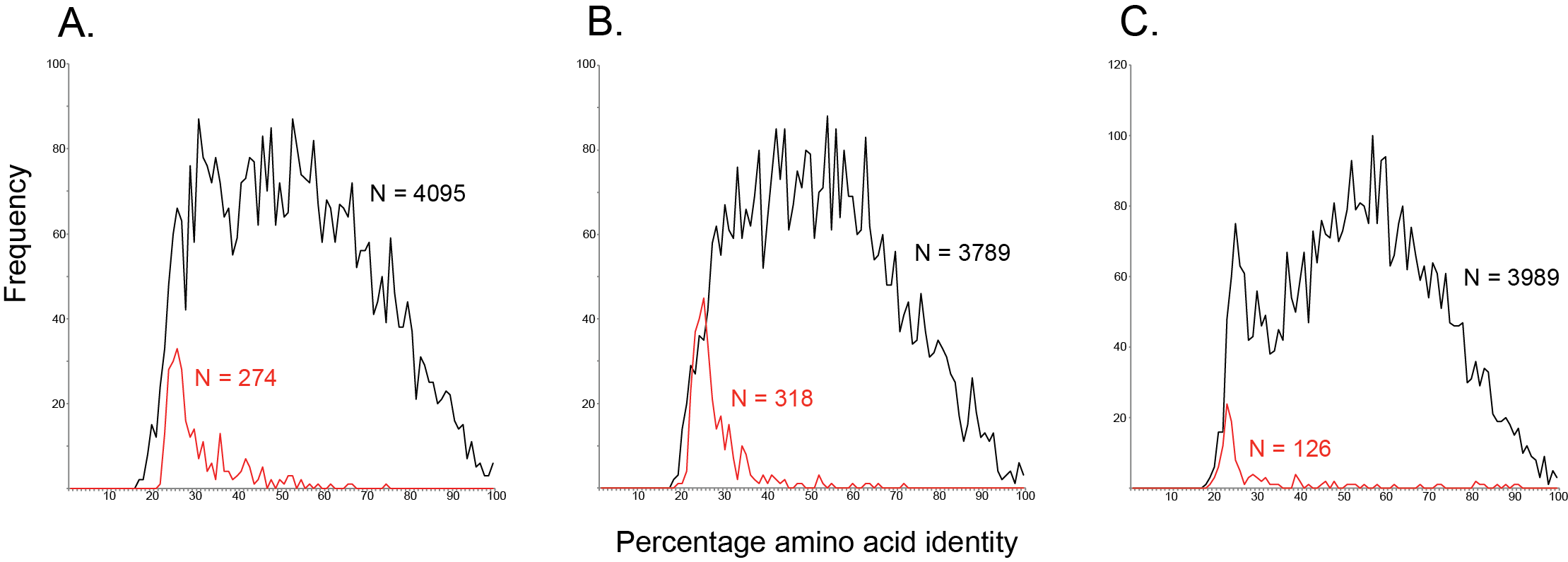
**

**Figure S2**. **Frequency distribution of percentage amino acid identity in genomic comparisons**. BLASTp found the best hit of each *B. bigemina* predicted protein in *B. bovis* (**A**.); each *B. divergens* predicted protein in *B. bovis* (**B**.) and each *B. divergens* predicted protein in *B. bigemina* (**C**.). Percentage amino acid identity of the best hits for VESA (red line) and all other proteins (black line) all VESA (red lines) are plotted in frequency distributions.

**
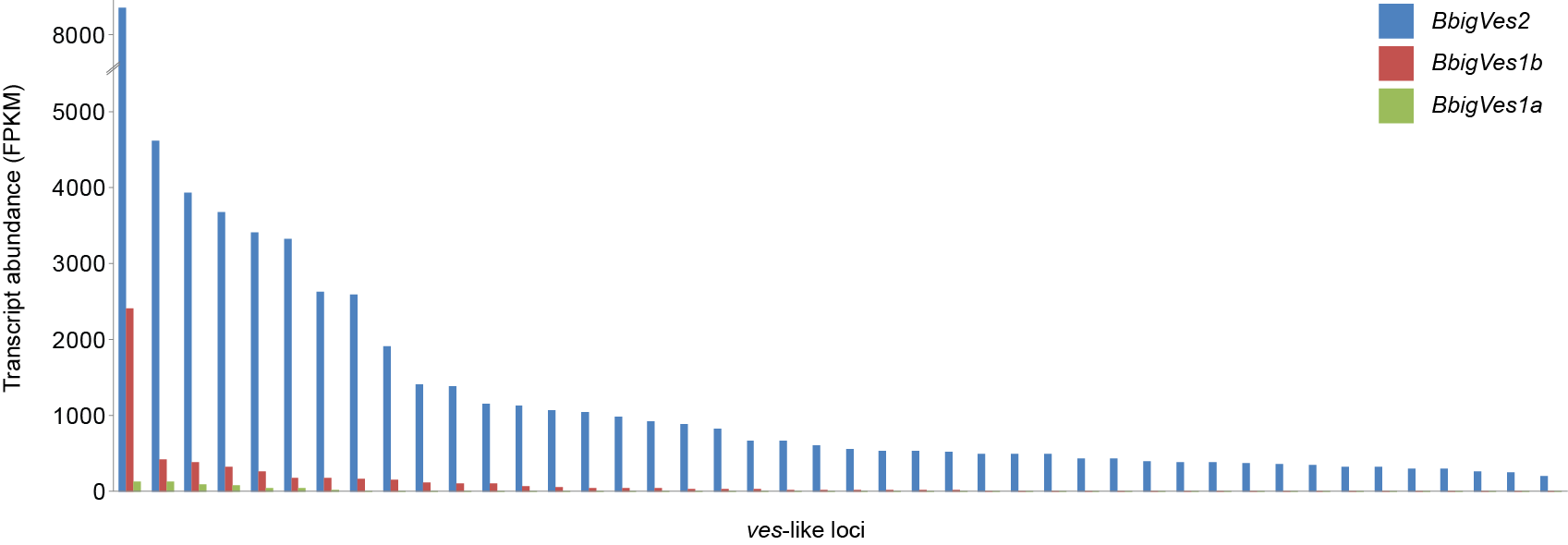
**

**Figure S3. Transcript abundance for *ves*-like genes in *B. bigemina* PR.** Transcript abundance, measured in Fragments Per Kilobase of transcript per Million fragments mapped (FPKM), was estimated for all *ves*-like genes after mapped RNA-seq data on to the *B. bigemina* PR genome. *Ves1* and *ves2* transcripts are ranked in order of decreasing abundance.

**
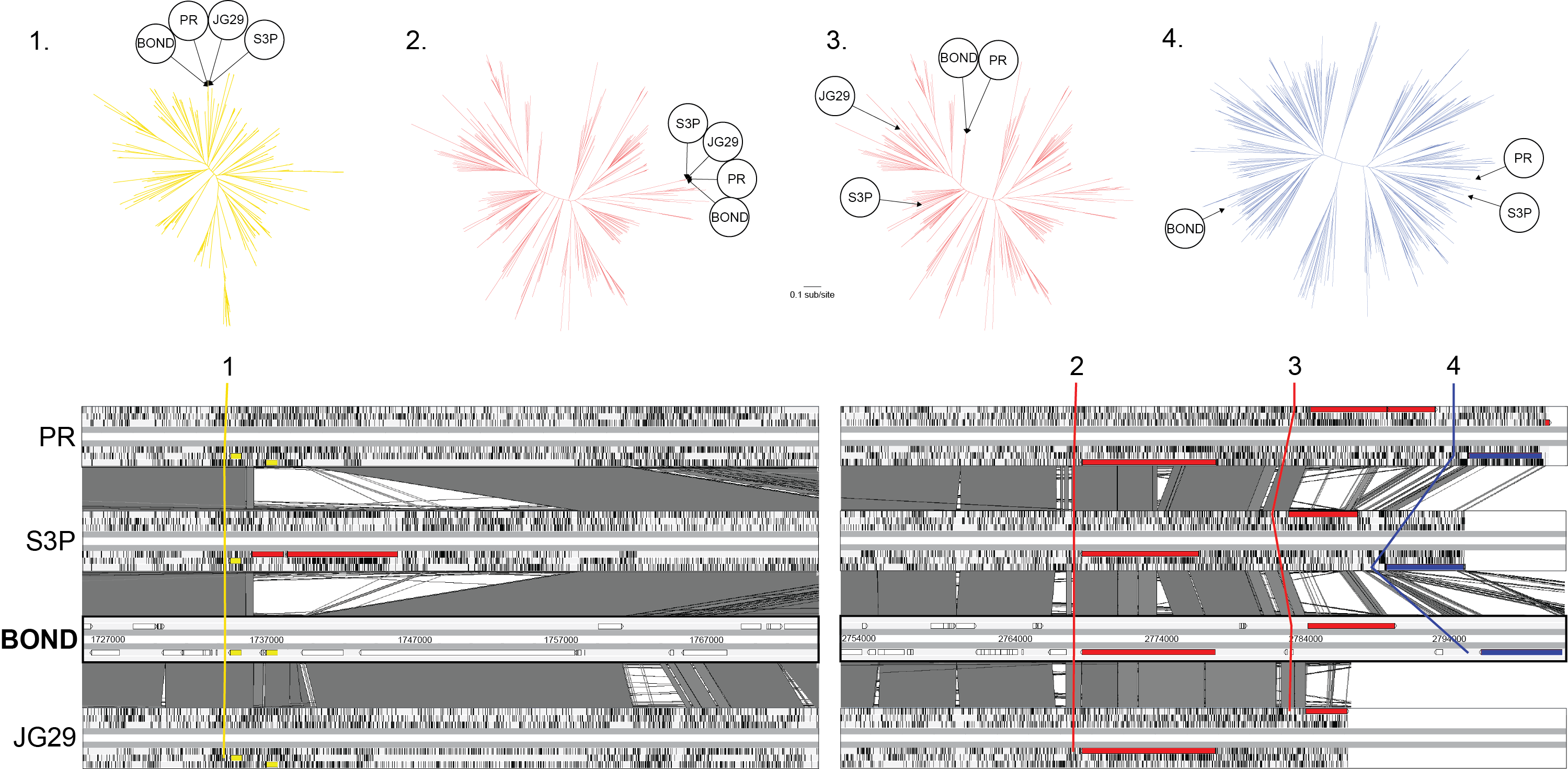
**

**Figure S4**. **Micro-homology surrounding *ves*-like loci in *B. bigemina***. Chromosome 2 sequences are shown for four *B. bigemina* strains as in Figure S1. The left panel shows two *ves2* loci (shaded yellow); the upstream gene is conserved in all strains (1), while the second gene is replaced in strain BbiS3P by a *ves1a* gene (shaded red). The right panel shows three *ves1* loci in tandem (2-4) at the chromosome right-hand end. Loci 2 and 3 contain *ves1a* genes (shaded red) while locus 4 contains a *ves1b* gene (shaded blue). Above the chromosomal sequences are unrooted, maximum likelihood phylogenetic trees for all *BbigVes2* (1), *BbigVes1a* (2-3) and *BbigVes1b* (4) gene sequences, estimated using a GTR+Γ model in PHYML. The positions of the genes portrayed in chromosomal comparisons are indicated on the trees by the black arrows. All trees are drawn to the same scale.


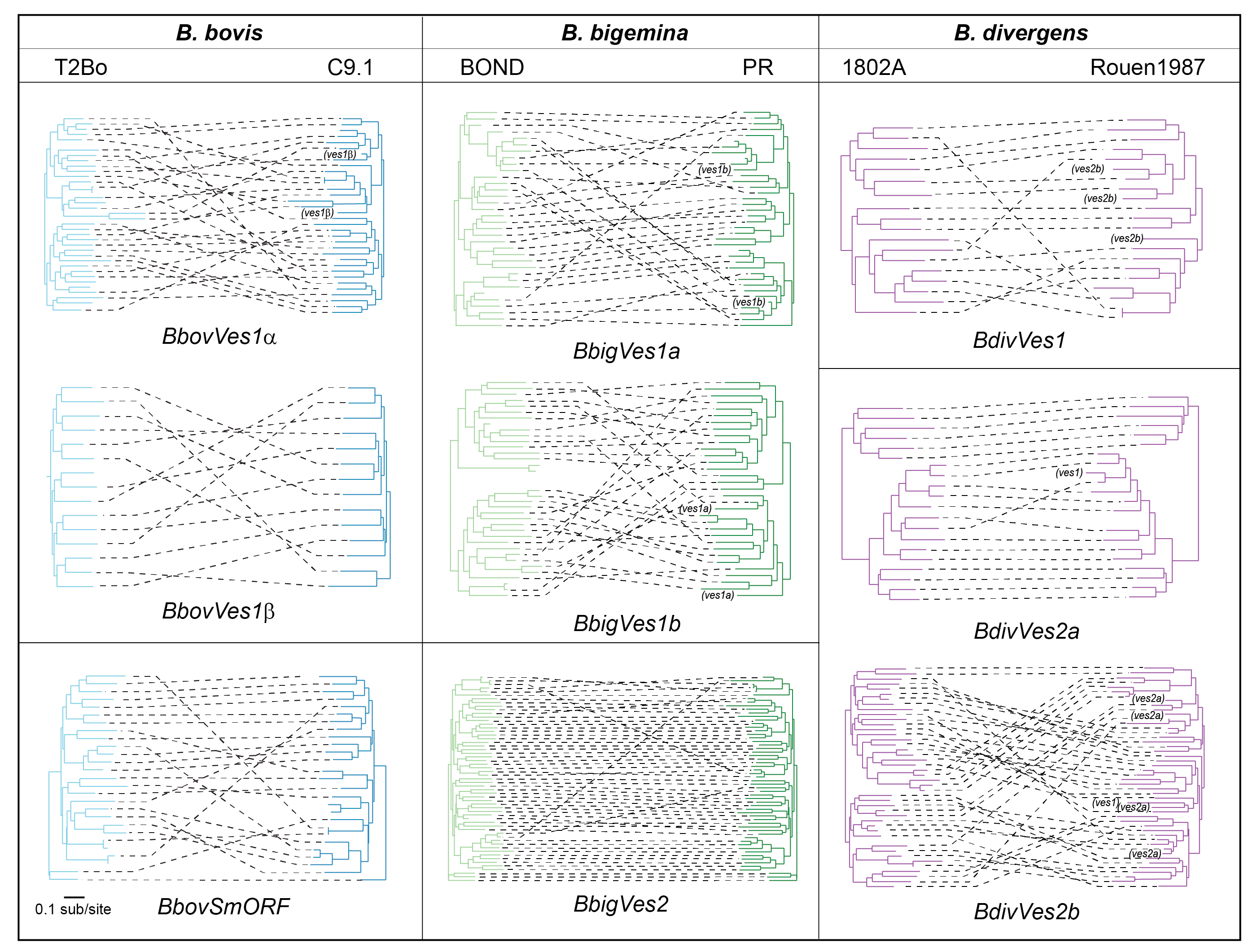


**Figure S5**. **Co-phylogenetic analysis of *ves*-like gene sequences in *Babesia* spp.** For each *ves1* and *ves2* gene family a tanglegram is shown. These comprise *ves* phylogenies for genes in the reference strain and one other strain (indicated at top) that are conserved in position in both genomes. Dashed lines connect terminal nodes that correspond to the same genomic position. In the absence of recombination, these two trees should be congruent, i.e. have the same topology; recombination, observed as transposition of a sequence between loci, results in incongruence. Where a gene from a different *ves* sub-family has been transposed into the locus, this is indicated in parentheses. The significance of congruence between the trees was assessed through permutation in each case; the results of these tests are shown in Figure 6.


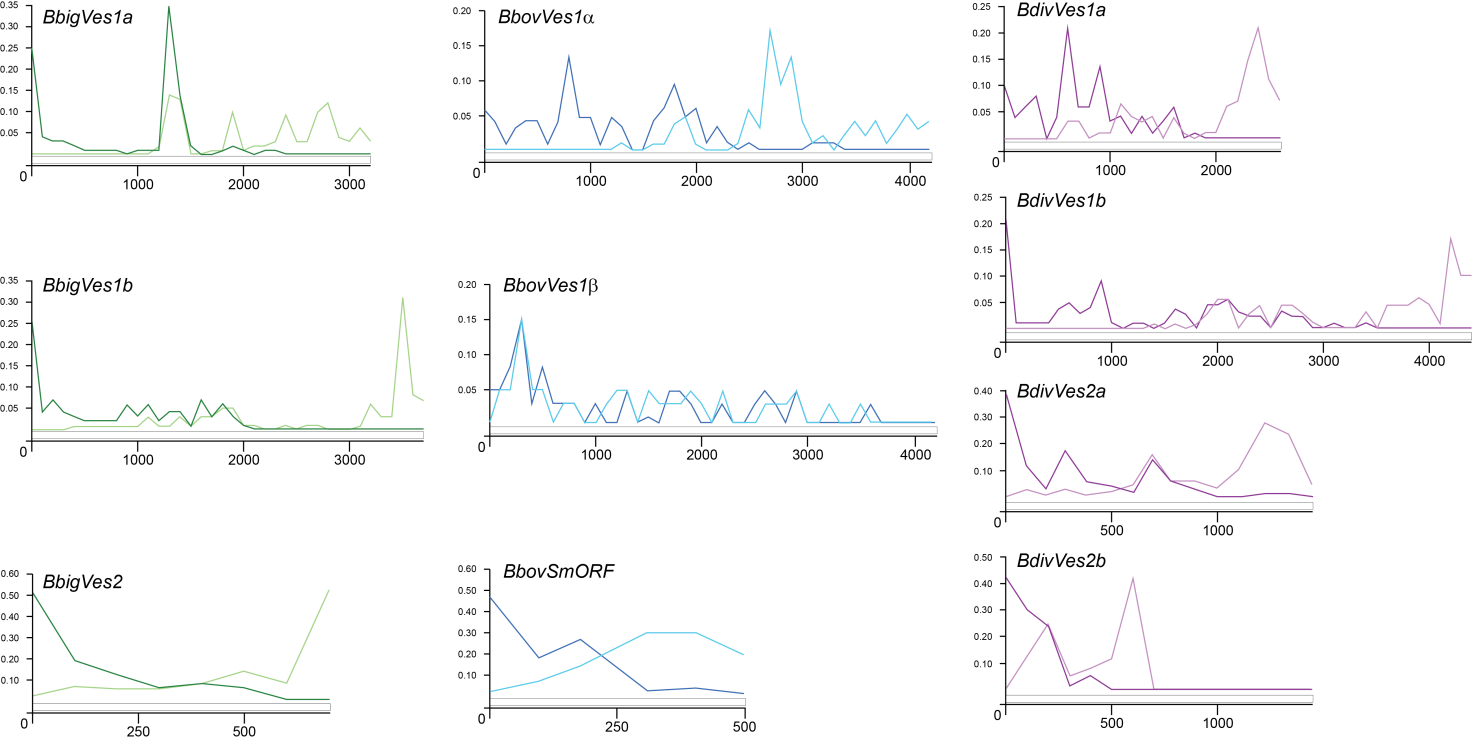


**Figure S6**. **Frequency of recombination breakpoints by position in *ves*-like sequence alignments**. For each sub-family, two lines are plotted along the nucleotide sequence alignment, corresponding to the frequency of 5’ (dark line) and 3’ (light line) breakpoints inferred by 3seq.
